# Pannexin 3-mediated adenosine triphosphate release from pre-osteoblasts promotes osteoblast differentiation through P2X4 receptor activation

**DOI:** 10.3389/fphys.2026.1880201

**Published:** 2026-09-21

**Authors:** Hiroaki Kito, Ayano Niwa, Takusei Ryu, Yohei Yamaguchi, Junko Kajikuri, Susumu Ohya

**Affiliations:** Department of Pharmacology, Graduate School of Medical Sciences, Nagoya City University, Nagoya, Japan

**Keywords:** ATP, differentiation, osteoblast, P2X4, Pannexin 3

## Abstract

Increased pre-osteoblast cell density within bone resorption areas is a key factor in promoting osteoblast differentiation; however, the molecular mechanisms underlying this process remain poorly understood. In this study, we investigated the role of ATP release and purinergic receptor P2X4 signaling in osteoblast differentiation using the murine pre-osteoblastic cell line MC3T3-E1. Under high cell density conditions, which were essential for the induction of osteoblast differentiation, we observed a significant accumulation of extracellular ATP released via the hemichannel Pannexin 3 (Panx3). This ATP accumulation subsequently activated the P2X4. Pharmacological inhibition and siRNA-mediated knockdown Panx3 markedly reduced extracellular ATP levels and decreased expression and activity of alkaline phosphatase (ALP), an osteoblast differentiation marker. Similarly, pharmacological inhibition and siRNA-mediated knockdown of P2X4 suppressed ALP expression and activity. Functional analyses using calcium imaging and whole-cell patch-clamp recording revealed that P2X4 activity was enhanced during osteoblast differentiation. Notably, this functional upregulation correlated with enhanced localization of P2X4 to the plasma membrane. Moreover, pharmacological inhibition of Panx3 or P2X4 impaired the endochondral ossification of isolated mouse metatarsal bone. These findings suggest that Panx3-mediated ATP release and the subsequent activation of P2X4 signaling constitute a crucial pathway linking cell density to osteoblast differentiation. Our study provides new insights into the purinergic signaling mechanisms underlying bone formation and identifies potential molecular targets for promoting osteogenesis.

## 1 Introduction

Bone remodeling is orchestrated by the coordinated expansion, differentiation, and activity of bone-resorbing osteoclasts and bone-forming osteoblasts. Recent studies have highlighted that osteoblast lineage cells expand as remodeling progresses (Andersen et al., 2013; Lassen et al., 2017). Osteoblasts are derived from mesenchymal stem cells and differentiate through three developmental stages: 1) cell expansion, 2) extracellular matrix secretion and maturation, and 3) matrix mineralization. In the second stage, immature osteoblasts differentiate into mature osteoblasts, which promote extracellular matrix maturation (Amarasekara et al., 2021). The initiation of osteoblast differentiation is triggered when pre-osteoblasts attain a critical cell density, whereas insufficient pre-osteoblast density markedly impairs or prevents osteoblast differentiation (Lassen et al., 2017). Consistent with this, previous studies have demonstrated that an optimal cell density of pre-osteoblasts is required for osteoblast differentiation (Bitar et al., 2008; Wang et al., 2023). This dependence on cell density strongly suggests that osteoblast differentiation requires direct cell–cell communication (e.g., via N-cadherin and connexin 43; Cx43) (Ferrari et al., 2000; Zappala et al., 2023), accumulation of secreted paracrine and autocrine factors (e.g., Wnt and bone morphogenic protein) (Martinovic et al., 2006; Lin and Hankenson, 2011), and/or mechanotransduction via the extracellular matrix (e.g., integrin β1) (Sun et al., 2018). However, the detailed mechanism by which increased cell density promotes osteoblast differentiation remains unclear.

Adenosine 5′-triphosphate (ATP) functions as a humoral signaling molecule in many tissues, including bone. Extracellular ATP release from osteoblasts is stimulated by mechanical loading (Romanello et al., 2001) and osteoblast maturation (Orriss et al., 2009). ATP release occurs via multiple mechanisms, including vesicular exocytosis (Orriss et al., 2009) and efflux through hemichannels formed by connexins or pannexins (Mikolajewicz et al., 2018). Pannexins (Panx1, Panx2, and Panx3) are a family of ATP-permeable membrane channel proteins that contribute to extracellular ATP signaling. Although these isoforms share structural similarities, they exhibit distinct tissue distributions and functional properties. Panx1 is broadly expressed and is widely implicated in purinergic signaling, whereas Panx2 is predominantly expressed in the central nervous system and has been associated with neuronal functions (Makarenkova et al., 2018). Panx3 is expressed in various tissues, including skeletal muscle, mammary glands, the male reproductive system, the cochlea, blood vessels, the small intestine, and the vomeronasal organs. Panx3 is particularly abundant in mineralized or hard tissues such as cartilage, bone, and teeth, where it is thought to regulate tissue development and homeostasis (O'Donnell and Penuela, 2021). Moreover, Panx3 has been shown to regulate osteoblast differentiation through multiple mechanisms, including a hemichannel and an endoplasmic reticulum Ca²^+^ channel (Ishikawa et al., 2011). Among the mechanisms by which Panx3 regulates osteoblast differentiation, it has been proposed that ATP released through Panx3 hemichannels may activate purinergic receptors on the osteoblast membrane via autocrine and paracrine signaling pathways.

Purinergic receptors of the P2X family are ligand-gated cation channels that translate extracellular ATP into cellular responses. Purinergic signaling influences the proliferation, differentiation, and function of bone-related cells such as osteoblasts, osteoclasts, and osteocytes (Carluccio et al., 2020; Zhou et al., 2021). Indeed, several studies have reported that extracellular ATP stimulates osteoblast differentiation (Cutarelli et al., 2016; Stovall et al., 2020). However, our understanding of purinergic receptor dynamics and function in osteoblast lineage cells remains limited (Nakamura et al., 2000; Rodrigues-Ribeiro et al., 2015), and the physiological role of ATP signaling in lineage progression has yet to be firmly established. The mRNA expression of several purinergic receptors, including P2X1–7 and multiple P2Y subtypes (Ferrari et al., 2000; Bitar et al., 2008; Orriss et al., 2009; Andersen et al., 2013; Lassen et al., 2017; Makarenkova et al., 2018; Mikolajewicz et al., 2018), has been detected in mature osteoblasts (Orriss et al., 2012). Previous studies have indicated a potential role for the P2X7 and P2X4 receptors in osteoblast differentiation; however, their direct functional properties as ion channels remain uncharacterized in these cells (Bergamin et al., 2021; Orriss et al., 2023).

Previous studies have demonstrated that Panx1-dependent ATP release functions as a key autocrine signal that activates P2X4/P2X7 receptors in TCR-stimulated T cells (Brock et al., 2022) and P2X1/P2X4 receptors at the immune synapses of activated T cells (Woehrle et al., 2010). Recently, it has been reported that extracellular ATP released by bone marrow osteoblasts via Panx3 activates P2X4 receptors in bone marrow plasma cells, thereby contributing to the maintenance of serum antibody titers (Ishikawa et al., 2024). Although functional interactions between pannexins and P2X receptors have been reported in various cell types, their functional relationship in osteoblasts remains insufficiently explored.

Despite evidence that Panx3-mediated ATP release can modulate P2X receptor activity, the precise mechanisms by which this signaling axis regulates osteoblast differentiation remain unclear. In particular, the functional role of P2X4 receptors in this process has yet to be fully elucidated. In the present study, we aimed to provide electrophysiological evidence characterizing P2X4 receptors as functional ligand-gated cation channels in osteoblasts. Using murine pre-osteoblastic MC3T3-E1 cells, murine bone marrow stromal cells, and murine fetal metatarsal organ cultures, we investigated the functional interplay between Panx3 and P2X4 receptors to elucidate how the Panx3/P2X4 signaling axis drives the progression of osteoblast differentiation.

## 2 Material and methods

### 2.1 Cell culture

The murine pre-osteoblast cell line MC3T3-E1 (RIKEN BRC, Tsukuba, Japan) was maintained at 37 °C in 5% CO_2_ with MEMα (FUJIFILM Wako Pure Chemical Corporation, Osaka, Japan) containing 10% fetal bovine serum (Sigma-Aldrich, St. Louis, MO, USA) and 1% penicillin/streptomycin mixture (hereafter referred to as normal medium). Osteoblastic differentiation of MC3T3-E1 cells was induced by the addition of ascorbic acid (50 μg/mL, Sigma-Aldrich), β-glycerophosphate (10 mM, Sigma-Aldrich), and dexamethasone (10 nM, FUJIFILM Wako Pure Chemical) to the normal medium (hereafter referred to as differentiation medium) (Kushibiki et al., 2015; Huang et al., 2024). Differentiating MC3T3-E1 cells were cultured in 24-well plates at a density of 5 × 10^4^ cells per well (high cell density required for the induction of osteoblast differentiation). For osteogenic differentiation, the culture medium was replaced with differentiation medium 24 h after cell seeding, and this time point was defined as day 0. The cells were cultured in differentiation medium for 7 days, with the medium changed every 2–3 days.

Mouse bone marrow stromal cells (BMSCs) were isolated from the femurs and tibias of 5-week-old male C57BL/6J mice as previously reported (Amend et al., 2016). BMSCs were cultured and induced to differentiate into bone marrow–derived osteoblasts in differentiation medium at 37 °C in a humidified incubator containing 5% CO_2_. Differentiating BMSCs were cultured in 24-well plates at a density of 1 × 10^5^ cells per well (high cell density required for the induction of osteoblast differentiation). For osteogenic differentiation, the culture medium was replaced with differentiation medium 24 h after cell seeding, and this time point was defined as day 0. The cells were cultured in differentiation medium for 7 days, with the medium changed every 2–3 days.

In this study, “undifferentiated” refers to cells collected at day 0 before osteogenic differentiation. “Differentiating” refers to cells cultured in differentiation medium for 7 days, whereas “normal” refers to cells cultured for the same period in normal medium without induction of differentiation.

### 2.2 Isolation and culture of murine embryonic metatarsals

Murine embryonic metatarsals were isolated and cultured as described previously (Houston et al., 2016). Briefly, the third metatarsal was dissected from E15.5 C57/BL6J mice and cultured in 24-well plates with 500 μL differentiation medium per well at 37 °C under a humidified 5% CO_2_ atmosphere for 7 days. Images of the metatarsals were acquired daily throughout the 7-day culture period. Total and mineralized areas of the metatarsals were measured by grayscale intensity analysis using ImageJ software v1.53t (NIH, Bethesda, MD, USA).

### 2.3 Transfection of small interference RNA and plasmid DNA

MC3T3-E1 cells were transiently transfected with Panx3 (s101771), Cx43 (s66668), and P2X4 (s71185) Silencer Select pre-designed siRNAs (20 nM) (Thermo Fisher Scientific, Waltham, MA, USA) in 24-well plates using Lipofectamine RNAiMAX (Thermo Fisher Scientific). Silencer Select Negative Control No.1 siRNA was used as the control.

Restriction enzyme-digested DNA fragments of Panx3 (GenBank accession number NM_172454) were ligated into the mammalian expression vector pcDNA3.1/Neo(+) (Thermo Fisher Scientific) using the TaKaRa ligation kit Ver. 1 (TaKaRa, Shiga, Japan). Cloned inserts were confirmed by DNA sequencing (Eurofins Genomics, Tokyo, Japan). MC3T3-E1 cells were transiently transfected with Panx3-expressing vector (250 ng/well) in 24-well plates using Lipofectamine 3000 (Thermo Fisher Scientific). An empty (mock) plasmid was used as a control for Panx3 expression plasmid transfection.

For osteogenic differentiation experiments, siRNA and/or plasmid DNA transfection was performed 24 h after cell seeding. Twenty-four hours after transfection, the normal medium was replaced with differentiation medium, and this time point was defined as day 0. The cells were then cultured in differentiation medium for 7 days, with the medium changed every 2–3 days.

### 2.4 RNA extraction and real-time PCR

Total RNA was extracted from the cells using the conventional acid guanidinium thiocyanate-phenol-chloroform method. Reverse transcription was performed using random hexamer primers (dN6; Roche, Basel, Switzerland) and RevaTra Ace RNase H reverse transcriptase (Toyobo, Osaka, Japan). Real-time PCR was conducted using SYBR Green assays on a QuantStudio 12 K Flex system (Applied Biosystems, Foster City, CA, USA). The expression levels of target mRNAs were normalized to those of GAPDH mRNA. The following primers were used for real-time PCR analysis: alkaline phosphatase (Alpl) (GeneBank accession number NM_007431, 287-396, 110 bp), bone sialoprotein (BSP) (NM_008318, 452-553, 102 bp), Panx1 (NM_019482, 539–638, 100 bp), Panx2 (NM_001002005, 736–835, 100 bp), Panx3 (NM_172454, 263–362, 100 bp), Cx43 (NM_010288, 481–580, 100 bp), P2X1 (NM_008771, 1194–1293, 100 bp), P2X2 (NM_001310700, 452–551, 100 bp), P2X3 (NM_145526, 942–1042, 101 bp), P2X4 (NM_011026, 475–574, 100 bp), P2X5 (NM_001376983, 1207–1306, 100 bp), P2X6 (NM_011028, 690–789, 100 bp), P2X7 (NM_011027, 989–1088, 100 bp), P2Y1 (NM_008772, 614–714, 99 bp), P2Y2 (NM_008773, 158–294, 137 bp), P2Y4 (NM_020621, 1589–1678, 90 bp), P2Y6 (NM_183168, 295–384, 90 bp), P2Y12 (NM_027571, 423–513, 91 bp), P2Y13 (NM_028808, 1455–1544, 90 bp), P2Y14 (NM_133200, 224–371, 148 bp), ANK (NM_020332, 811–890, 80 bp), VNUT (NM_183161, 905–994, 90 bp), and GAPDH (NM_008084, 912–1022, 111 bp).

### 2.5 Western blotting

Whole-cell lysates and plasma membrane fractions were prepared from MC3T3-E1 cells. Protein concentrations were determined for both fractions using a protein assay kit (Bio-Rad Laboratories, Hercules, CA, USA) with bovine serum albumin as a standard. Plasma membrane fractions were prepared using the Trident Membrane Protein Extraction Kit (GeneTex, Irvine, CA, USA) according to the manufacturer’s instructions, and 10 μg of protein was loaded per lane for Western blot analysis. Proteins were separated by SDS-PAGE on 15% polyacrylamide gels and transferred to PVDF membranes. Membranes were incubated with rabbit anti-P2X4 polyclonal antibody (1:500, A6682, ABclonal, Woburn, MA, USA), rabbit anti-Na^+^/K^+^-ATPase polyclonal antibody (1:500, A7878, ABclonal), rabbit anti-FLOT2 polyclonal antibody (1:500, A6590, ABclonal), and mouse anti-GAPDH monoclonal antibody (1:1000, M171-3, MBL, Nagoya, Japan) overnight at 4 °C, followed by HRP-conjugated secondary antibodies (1:10000, Merck Millipore, Darmstadt, Germany) for 1 hr. Target protein bands around the predicted band sizes of P2X4 (62 kDa), Na^+^/K^+^-ATPase (110 kD), and GAPDH (37 kDa) were visualized using enhanced chemiluminescence reagent and captured using the Amersham Imager 600 (GE Healthcare Japan, Tokyo, Japan).

### 2.6 Confocal imaging

Undifferentiated (day 0) and differentiating (day 7) MC3T3-E1 cells were trypsinized to obtain a single-cell suspension, seeded onto glass-bottom dishes, and cultured for 12 h in normal or differentiation medium before immunostaining. Cells were washed three times with PBS and fixed with 4% paraformaldehyde in PBS for 10 min at room temperature. After fixation, the cells were permeabilized and blocked with 0.1% Triton X-100/3% bovine serum albumin in PBS for 10 min at room temperature. Cells were incubated with the primary antibody rabbit anti-P2X4 polyclonal antibody (1:200, A6682, ABclonal) for 6 h at 4 °C. After washing three times with PBS, the cells were incubated with an Alexa Fluor 488-conjugated secondary antibody (1:1000, A21206, Thermo Fisher Scientific) for 1 h at room temperature in the dark. Fluorescence images were visualized using a confocal laser scanning microscope system A1R (Nikon, Tokyo, Japan). Image data were analyzed using ImageJ software v1.53t.

### 2.7 Alkaline phosphatase activity

MC3T3-E1 cells and BMSCs were seeded at densities of 5 × 10^4^ and 1 × 10^5^ cells per well, respectively, in 24-well plates and cultured in differentiation medium for 7 days. Cells were lysed with 300 μL of 0.1% Triton X-100 followed by three freeze–thaw cycles. Lysates were centrifuged at 15000 rpm for 15 min, and ALP activity in the supernatant was measured using the LabAssay ALP kit (FUJIFILM Wako Pure Chemical) according to the manufacturer’s instructions.

### 2.8 Measurement of extracellular ATP level

To measure extracellular ATP release, MC3T3-E1 cells were seeded at a density of 5 × 10^4^ cells per well in 24-well plates and cultured in differentiation medium for 7 days. After 7 days of differentiation, the cells were washed with PBS, and the medium was replaced with fresh differentiation medium with or without CBX (100 μM). For experiments involving siRNA or plasmid DNA, the cells were washed after 7 days of differentiation, and the medium was replaced with fresh differentiation medium. In both experiments, the cells were incubated for 1 h. The conditioned medium was then collected, and the ATP concentration was measured using a luciferin–luciferase-based assay. Briefly, 100 μL of conditioned medium was transferred to a white 96-well plate and mixed with an equal volume of CellTiter-Glo 2.0 reagent (Promega, Madison, WI, USA). The mixture was incubated for 2 min, and luminescence was measured using a microplate luminometer to determine extracellular ATP levels.

### 2.9 Alizarin red S staining

MC3T3-E1 cells and BMSCs were cultured for 7 days in 24-well plates in differentiation medium. Cells were fixed with 2% paraformaldehyde and stained with 2% (w/v) Alizarin Red S solution (FUJIFILM Wako Pure Chemical). Samples were washed three times with distilled water, and bright-field images were acquired using an Axio Observer Z1 microscope (Zeiss, Oberkochen, Germany). Calcified nodules were solubilized in 5% formic acid, and calcium deposition was quantified by measuring the absorbance of the solution at 450 nm.

### 2.10 Measurement of intracellular Ca^2+^ concentration

Measurement of [Ca^2+^]_i_ was performed as previously described (Kito et al., 2023). Briefly, MC3T3-E1 cells were loaded with 10 μM Fura-2 acetoxymethyl ester (Fura-2 AM; Dojindo, Kumamoto, Japan) in a standard HEPES solution for 40 min at room temperature. After washing for 10 min to remove excess Fura-2 AM, the fluorescence ratio (340/380 nm) was recorded every 2 s using the HCImage system (Hamamatsu Photonics, Shizuoka, Japan). The standard HEPES solution contained 137 mM NaCl, 5.9 mM KCl, 2.2 mM CaCl_2_, 1.2 mM MgCl_2_, 14 mM glucose, and 10 mM HEPES (pH 7.4, adjusted with NaOH).

### 2.11 Electrophysiology

Whole-cell patch-clamp recordings were performed as previously described (Kito et al., 2023). Briefly, whole-cell configuration was established on single MC3T3-E1 cells using the Axopatch 200B amplifier (Molecular Devices, San Jose, CA, USA). Data were acquired with a Digidata 1550B interface and analyzed using pCLAMP software v11.1 (Molecular Devices). Currents were low-pass filtered at 1 kHz and sampled at 10 kHz. Series resistance was compensated by 70%. Patch pipettes filled with the internal solution had resistances of 3–5 MΩ. A 200-ms voltage ramp from +40 mV to −120 mV was applied every 10 s from a holding potential of −10 mV. The external solution contained 137 mM NaCl, 5.9 mM KCl, 2.2 mM CaCl_2_, 1.2 mM MgCl_2_, 14 mM glucose, and 10 mM HEPES (pH 7.4, adjusted with NaOH). The internal (pipette) solution contained 140 mM CsCl, 4 mM MgCl_2_, 5 mM EGTA, 10 mM HEPES, and 2 mM Na_2_ATP (pH 7.2, adjusted with CsOH).

### 2.12 Reagents

The gap junction blocker 18β-glycyrrhetinic acid (18βGA; Sigma-Aldrich), the selective P2X4 inhibitors 5-BDBD (IC_50_: 2.5 μM; MedChemExpress, Monmouth Junction, NJ, USA) and PSB12062 (PSB; IC_50_: 1.4 μM; MedChemExpress), and the selective P2X7 inhibitor A804598 (IC_50_: 10 nM; MedChemExpress) were dissolved in dimethyl sulfoxide (DMSO) to prepare 10 mM stock solutions. In contrast, the hemichannel inhibitor carbenoxolone (CBX; TOCRIS Bioscience, Ellisville, MO, USA), the Cx43-mimetic inhibitory peptide Gap26 (Sigma-Aldrich), the Panx1-mimetic inhibitory peptide 10Panx (TOCRIS Bioscience), the non-selective P2X1/2/3/5 inhibitors PPADS (Cayman Chemical, Ann Arbor, MI, USA) and suramin (Sigma-Aldrich), and the P2X agonist BzATP (MedChemExpress) were dissolved in distilled water to prepare 10–100 mM stock solutions. ATP-hydrolyzing enzyme apyrase (Sigma-Aldrich) was dissolved in distilled water to prepare 1.0 mg/mL stock solutions. When compounds dissolved in water and DMSO were used in the same experiment, DMSO was defined as the vehicle, with the same final concentration in all treatment groups.

### 2.13 Statistical analysis

Pooled data are presented as the mean ± standard error of the mean (SEM). For comparisons between two groups, homogeneity of variance was assessed using an F-test, followed by an unpaired t-test. For comparisons among more than two groups, one-way analysis of variance (ANOVA) was performed, followed by the Tukey–Kramer *post hoc* test. A p-value < 0.05 was considered statistically significant. Significance levels of *P* < 0.05 and *P* < 0.01 are indicated in the figures.

## 3 Results

### 3.1 Identification of pannexin and P2X subtypes expressed in differentiating MC3T3-E1 cells

To clarify the time-dependent induction of osteoblast differentiation, we measured the mRNA expression levels of Alpl and BSP, osteoblast differentiation markers, in MC3T3-E1 cells at days 0, 7, 14, and 21 (**Figures 1A, B**). The expression of Alpl, an early differentiation marker, markedly increased from day 0 to day 14. In contrast, the expression of BSP, a late differentiation marker, showed a gradual increase. We also examined the mRNA expression of Runx2, a key transcription factor involved in osteoblast differentiation, under the same conditions. However, the expression levels of Runx2 mRNA did not differ significantly among days 0, 7, 14, and 21 (**Supplementary Figure S1**). MC3T3-E1 cells were cultured in differentiation medium from day 0 to day 21 and then stained with Alizarin Red S to visualize calcium deposition (**Figure 1C**). Quantitative analysis revealed a significant increase in calcium deposition at days 7, 14, and 21 compared with undifferentiated cells, although only minimal calcium deposition was visually detectable at day 21 (**Figure 1D**). The marked increase in Alpl mRNA expression at day 7 suggested that the signaling pathways involved in osteoblast differentiation were activated as early as day 7 after the induction of differentiation, without substantial mineral deposition at this stage. Therefore, we examined the mRNA expression of pannexin and P2X receptor subtypes at this time point. Quantitative real-time PCR analysis revealed that Panx3 and P2X4 were abundantly expressed in differentiating MC3T3-E1 cells at day 7 (**Figures 1E, F**). We also examined the expression of P2Y receptor subtypes reported to be expressed in osteoblasts, including P2Y1, P2Y2, P2Y4, P2Y6, P2Y12, P2Y13, and P2Y14 (Orriss et al., 2012). Although these P2Y receptor subtypes were detectable, their expression levels were markedly lower than that of P2X4 (**Figure 1G**).

**Figure 1:**
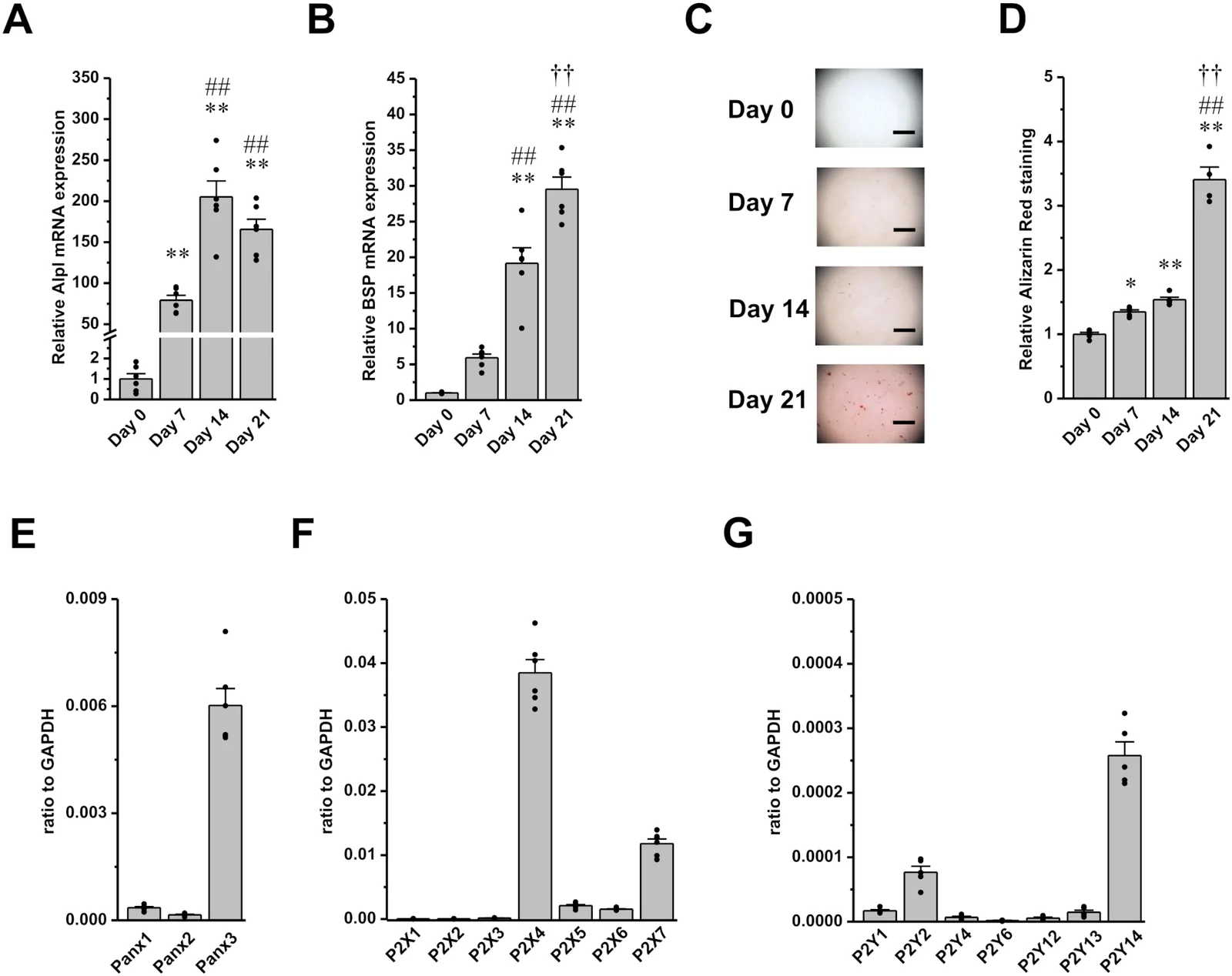
Molecular identification of pannexin and P2X in mouse pre-osteoblast MC3T3-E1 cells during osteoblast differentiation. **(A, B)** Real-time PCR for alkaline phosphatase (Alpl, **A**) and bone sialoprotein (BSP, **B**) in differentiating MC3T3-E1 (days 0, 7, 14, and 21) (n = 6 independent culture preparation per group, ** p < 0.01 vs. Day 0, ## p < 0.01 vs. Day 7, †† p < 0.01 vs. Day 14). The expression levels of Alpl and BSP mRNAs were normalized to the expression levels at day 0. **(C)** Representative images of Alizarin Red S staining in differentiating MC3T3-E1 cells (days 0, 7, 14, and 21). The scale bar is 500 μm. **(D)** Summarized data from experiments shown in panel C (n = 5 independent culture preparation per group, *;** p < 0.05; 0.01 vs. Day 0, ## p < 0.01 vs. Day 7, †† p < 0.01 vs. Day 14). **(E)** Real-time PCR for pannexin subtypes in differentiating MC3T3-E1 (day 7) (n = 6 independent culture preparation per group). The expression levels of pannexin mRNAs were normalized to that of endogenous GAPDH. **(F)** Real-time PCR for P2X subtypes in differentiating MC3T3-E1 (day 7) (n = 6 independent culture preparation per group). The expression levels of P2X mRNAs were normalized to that of endogenous GAPDH. **(G)** Real-time PCR for P2Y subtypes in differentiating MC3T3-E1 (day 7) (n = 6 independent culture preparation per group). The expression levels of P2Y mRNAs were normalized to that of endogenous GAPDH.

### 3.2 Contribution of Panx3 to osteoblast differentiation in MC3T3-E1 cells

To clarify the involvement of Panx3 in the early stage of osteoblast differentiation, we examined Alpl mRNA expression in differentiating MC3T3-E1 cells (day 7) treated with the hemichannel inhibitor CBX (10 or 100 μM), the gap junction inhibitor 18βGA (30 μM), the Cx43-mimetic inhibitory peptide Gap26 (300 μM), and the Panx1-mimetic inhibitory peptide 10Panx (100 μM). Treatment with 100 μM CBX markedly decreased Alpl mRNA expression in differentiating MC3T3-E1 cells. To determine whether this suppression was attributable to connexin inhibition by CBX, we examined the effect of 18βGA on Alpl mRNA expression. Treatment with 18βGA had no effect on Alpl mRNA expression in differentiating MC3T3-E1 cells. Although several reports have indicated that Cx43 is functionally expressed in osteoblasts (Stains et al., 2014), treatment with Gap26 did not alter Alpl mRNA expression. Furthermore, 10Panx did not affect Alpl mRNA expression (**Figure 2A**).

**Figure 2:**
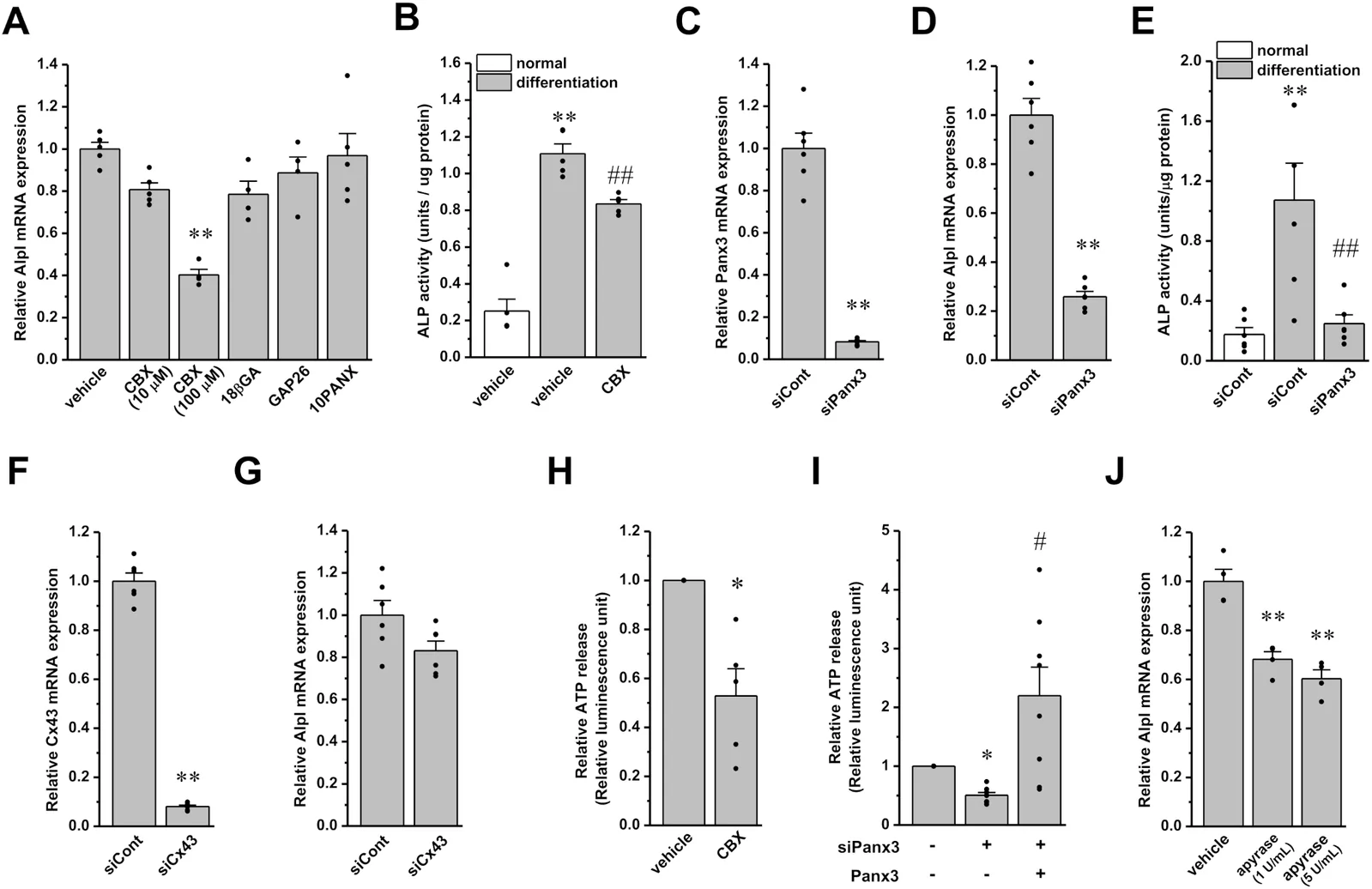
Involvement of Pannexin 3 in the osteoblast differentiation of MC3T3-E1 cells. **(A)** Real-time PCR for Alpl in differentiating MC3T3-E1 (day 7) treated with CBX (10 or 100 μM), 18βGA (30 μM), Gap26 (300 μM), and 10Panx (100 μM) for 7 days. The expression levels of Alpl mRNA were normalized to that in the vehicle control group (n = 5 for each group, ** p < 0.01 vs. vehicle). **(B)** ALP activity of MC3T3-E1 cells was measured in normal or differentiation medium in the absence or presence of CBX (100 μM) for 7 days (n = 5 for each, ** p < 0.01 vs. normal-vehicle, ## p < 0.01 vs. differentiation-vehicle). **(C, D)** Real-time PCR for Pannexin 3 (Panx3) and Alpl in differentiating MC3T3-E1 (day 7) transfected with Panx3 siRNA (siPanx3). The expression levels of Panx3 and Alpl mRNAs were normalized to those in cells transfected with control siRNA (siCont) (n = 6 for each, ** p < 0.01 vs. siCont). **(E)** ALP activity of MC3T3-E1 cells was measured in normal or differentiation medium in the absence or presence of siRNA-mediated knockdown of Panx3 for 7 days (n = 5 for each, ** p < 0.01 vs. normal-siCont, ## p < 0.01 vs. differentiation-siCont). **(F, G)** Real-time PCR for Connexin 43 (Cx43) and Alpl in differentiating MC3T3-E1 (day 7) transfected with Cx43 siRNA (siCx43). The expression levels of Cx43 and Alpl mRNAs were normalized to those in cells transfected with control siRNA (siCont) (n = 6 for each, ** p < 0.01 vs. siCont). **(H)** The effects of CBX (100 μM) on ATP release from differentiating MC3T3-E1 cells (day 7). After 7 days of osteogenic differentiation, cells were washed and incubated in fresh differentiation medium with or without CBX (100 μM) for 1 h. The conditioned medium was collected, and extracellular ATP levels were measured using a luciferin–luciferase-based assay. ATP levels were normalized to those in the vehicle control group (n = 5 for each, * p < 0.05 vs. vehicle). **(I)** Extracellular ATP levels in differentiating MC3T3-E1 cells (day 7) following Panx3 siRNA treatment and Panx3 expression plasmid transfection. Cells were transfected with control siRNA plus empty vector, Panx3 siRNA plus empty vector, or Panx3 siRNA plus a Panx3 expression plasmid. After 7 days of osteogenic differentiation, the cells were washed and incubated in fresh differentiation medium for 1 h. The conditioned medium was collected, and extracellular ATP levels were measured using a luciferin–luciferase-based assay. ATP levels were normalized to those in the control group (siPanx3^−^Panx3^−^) (n = 8 for each, * p < 0.05 vs. siPanx3^−^Panx3^−^, # p < 0.05 vs. siPanx3^+^Panx3^−^). **(J)** Real-time PCR for Alpl mRNA in differentiating MC3T3-E1 (day 7) treated with apyrase (1 and 5 U/mL) for 7 days. The expression level of Alpl mRNA was normalized to that in the vehicle control group (n = 4 for each, ** p < 0.01 vs. vehicle).

Alpl is the gene encoding alkaline phosphatase (ALP). To assess whether changes in Alpl mRNA expression correlate with ALP enzyme activity, ALP activity was measured in differentiating MC3T3-E1 cells. ALP activity was significantly higher in differentiating MC3T3-E1 cells (day 7) (vehicle) than in normal MC3T3-E1 cells (day 7) (vehicle). Treatment with CBX (100 μM) significantly reduced ALP activity in differentiating MC3T3-E1 cells (**Figure 2B**).

Next, we examined whether siRNA-mediated knockdown of Panx3 inhibited osteoblast differentiation. Panx3 mRNA expression levels were markedly reduced following transfection with Panx3 siRNA (**Figure 2C**). To assess the selectivity of Panx3 siRNA, we analyzed potential off-target effects by measuring the expression levels of Panx1 and Panx2 mRNA in differentiating MC3T3-E1 cells (**Supplementary Figure S2A**). Consistent with pharmacological inhibition, Panx3 knockdown significantly reduced Alpl mRNA expression (**Figure 2D**) and ALP activity (**Figure 2E**). In contrast, siRNA-mediated knockdown of Cx43 markedly reduced Cx43 mRNA expression but had no significant effect on Alpl mRNA expression in differentiating MC3T3-E1 cells (**Figures 2F, G**).

Ishikawa et al. reported that Panx3 released intracellular ATP through its hemichannel activity in Panx3-overexpressing C2C12 cells (Ishikawa et al., 2019). Therefore, we examined whether endogenous Panx3 hemichannels contributed to ATP release from MC3T3-E1 cells. ATP release was significantly suppressed by 100 μM CBX in differentiating MC3T3-E1 cells (**Figure 2H**). In addition, Extracellular ATP levels were reduced in Panx3 knockdown MC3T3-E1 cells following 7 days of osteogenic differentiation. Importantly, Panx3 overexpression significantly rescued the reduction in ATP release from Panx3-knockdown cells (**Figure 2I**), demonstrating that Panx3 positively regulates ATP release during osteoblast differentiation. In addition, treatment with ATP-hydrolyzing enzyme apyrase (1 or 5 U/mL) significantly suppressed Alpl mRNA expression in differentiating MC3T3-E1 cells (**Figure 2J**).

### 3.3 Contribution of P2X4 to osteoblast differentiation in MC3T3-E1 cells

To clarify the involvement of P2X4 in the early stage of osteoblast differentiation, we examined Alpl mRNA expression in differentiating MC3T3-E1 cells (day 7) treated with the specific P2X4 inhibitors 5-BDBD (5 or 10 μM) and PSB (5 μM), the P2X7 inhibitor A804598 (10 μM), and the non-selective P2X inhibitors PPADS (10 μM) and suramin (10 μM). Treatment with the P2X4 inhibitors 5-BDBD or PSB significantly suppressed Alpl mRNA expression in differentiating MC3T3-E1 cells. In contrast, treatment with PPADS or suramin had no effect on Alpl mRNA expression (**Figure 3A**). PPADS and suramin (10 µM) are expected to inhibit P2X1, P2X2, P2X3, and P2X5, while having limited effects on P2X4, for which their IC_50_ values are >500 and >300 µM, respectively (Illes et al., 2021).

**Figure 3:**
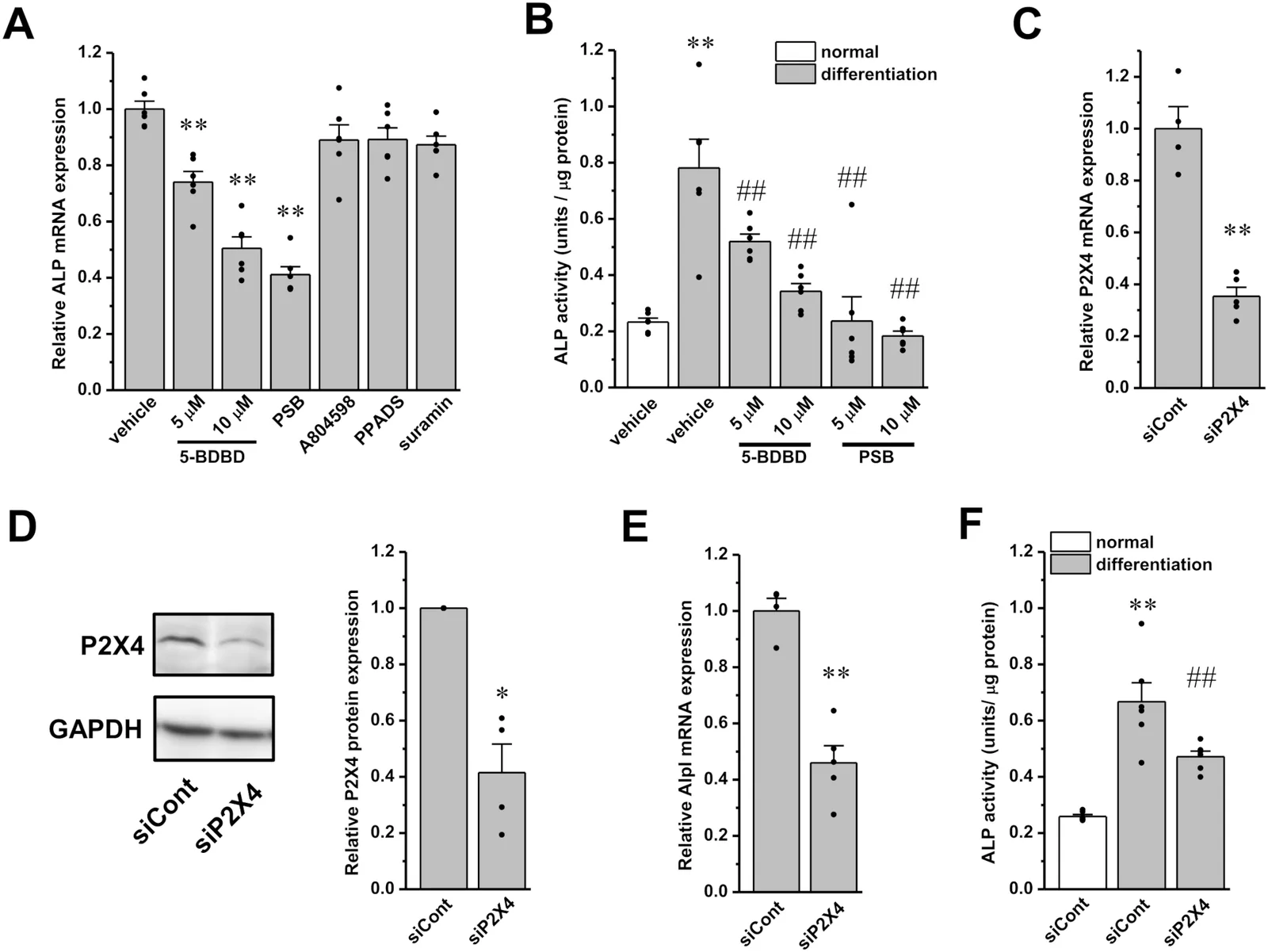
Involvement of P2X4 in the osteoblast differentiation of MC3T3-E1 cells. **(A)** Real-time PCR for Alpl in differentiating MC3T3-E1 (day 7) treated with 5-BDBD (5 or 10 μM), PSB (5 μM), A804598 (10 μM), PPADS (10 μM) and suramin (10 μM) for 7 days. The expression level of Alpl mRNA was normalized to that in the vehicle control group (n = 5 for each group, ** p < 0.01 vs. vehicle). **(B)** ALP activity of MC3T3-E1 cells was measured in normal or differentiation medium with 5-BDBD (5 and 10 μM) or PSB (5 and 10 μM) for 7 days (n = 5 for each, ** p < 0.01 vs. normal-vehicle, ## p < 0.01 vs. differentiation-vehicle). **(C)** Real-time PCR for P2X4 in differentiating MC3T3-E1 (day 7) transfected with P2X4 siRNA (siP2X4). The expression level of P2X4 mRNA were normalized to that in control siRNA (siCont) group (n = 5 for each, ** p < 0.01 vs. siCont). **(D)** Whole cell protein lysates of differentiating MC3T3-E1 cells (day 7) transfected with siCont and siP2X4 were immunoblotted with anti-P2X4 (top) and anti-GAPDH (bottom) antibodies on the same filter. Data are summarized as the optical densities of P2X4 and GAPDH band signals. Protein expression of P2X4 in the siCont group was expressed as 1.0 after the optical density of the P2X4 signal was normalized to that of the GAPDH signal (n = 4 for each, * p < 0.05 vs. siCont). **(E)** Real-time PCR for Alpl in differentiating MC3T3-E1 (day 7) transfected with siP2X4. The expression level of Alpl mRNA was normalized to that in the siCont group (n = 5 for each, ** p < 0.01 vs. siCont). **(F)** ALP activity of MC3T3-E1 cells was measured in normal or differentiation medium in the absence or presence of siRNA-mediated knockdown of P2X4 for 7 days (n = 6 for each, ** p < 0.01 vs. normal-siCont, ## p < 0.01 vs. differentiation-siCont).

ALP activity was significantly higher in differentiating MC3T3-E1 cells (day 7) (vehicle) than in normal MC3T3-E1 cells (day 7) (vehicle). This increase in ALP activity was significantly reduced by treatment with 5-BDBD or PSB (5 or 10 μM) (**Figure 3B**).

Next, we examined whether siRNA-mediated knockdown of P2X4 inhibited osteoblast differentiation. P2X4 mRNA and protein expression levels were markedly reduced following transfection with P2X4 siRNA (**Figures 3C, D**). Off-target effects were assessed by measuring the expression levels of P2X1–P2X7 mRNAs (**Supplementary Figure S2B**). A significant decrease in Alpl mRNA expression was observed in differentiating MC3T3-E1 cells transfected with P2X4 siRNA (**Figure 3E**). The differentiation-associated increase in ALP activity compared with normal MC3T3-E1 cells was significantly suppressed by P2X4 siRNA transfection (**Figure 3F**).

### 3.4 Inhibitory effects of 5-BDBD on the BzATP-induced Ca^2+^ influx in differentiating MC3T3-E1 cells

Intracellular Ca²^+^ concentration ([Ca²^+^]_i_) changes in response to 100 μM BzATP were measured in MC3T3-E1 cells loaded with Fura-2 AM to assess the effects of P2X4 inhibition. Application of BzATP (100 μM) had no effect on [Ca²^+^]_i_ in undifferentiated MC3T3-E1 cells (day 0) (**Figure 4A**), whereas BzATP increased [Ca²^+^]_i_ in differentiating MC3T3-E1 cells cultured for 7 days (**Figure 4B**). Although BzATP is commonly used as a P2X7 agonist, it is also known to activate P2X4 receptors. In this study, BzATP was used to preferentially stimulate P2X4, as reported EC_50_ values for mouse P2X receptor subtypes are 24 μM for P2X4 and 285 μM for P2X7 (Young et al., 2007; Fortuny-Gomez and Fountain, 2024).

**Figure 4:**
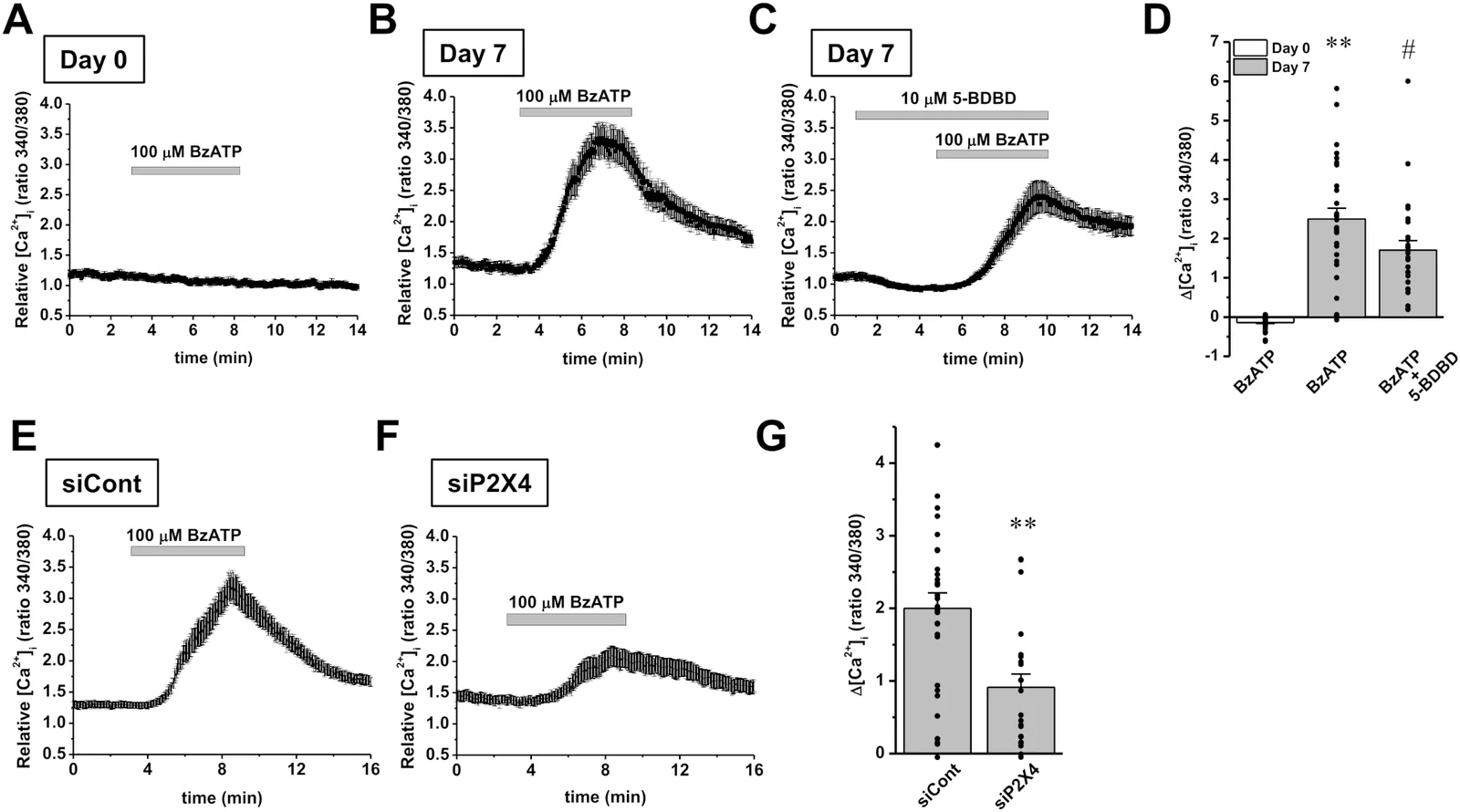
Contribution of P2X4 to Ca^2+^ influx in differentiating MC3T3-E1 cells. **(A, B)** Recordings of [Ca^2+^]_i_ responses to 100 μM BzATP were obtained in differentiating MC3T3-E1 cells (day 0; **A**, day 7; **B**). Data represent mean traces ± SEM. **(C)** Recordings of [Ca^2+^]_i_ changes by the co-treatment with 10 μM 5-BDBD and 100 μM BzATP were obtained in differentiating MC3T3-E1 cells (day 7). Data represent mean traces ± SEM. **(D)** Data regarding the BzATP-induced increases in [Ca^2+^]_i_ obtained from the experiments shown in **(A–C)** are summarized. The peak amplitude of the increase in [Ca^2+^]_i_ was measured in differentiating MC3T3-E1 cells (day 0 and 7) (Day 0-BzATP: n = 37, Day 7-BzATP: n=27; Day 7-BzATP + 5-BDBD: n=26, ** p < 0.01 vs. Day 0-BzATP, # p < 0.05 vs. Day 7-BzATP). **(E, F)** Recordings of [Ca^2+^]_i_ changes by the addition of 100 μM BzATP were obtained in differentiating MC3T3-E1 cells (day 7) transfected with siCont **(E)** and siP2X4 **(F)**. Data are presented as the mean trace ± SEM. **(G)** Data regarding the BzATP-induced increases in [Ca^2+^]_i_ obtained from the experiments shown in **(E, F)** are summarized. The peak amplitude of the increase in [Ca^2+^]_i_ was measured in siCont and siP2X4-transfected MC3T3-E1 cells (siCont: n = 30, siP2X4: n=22, ** p < 0.01 vs. siCont).

The BzATP (100 μM)-induced [Ca²^+^]_i_ increase was inhibited by treatment with 5-BDBD (10 μM) in differentiating MC3T3-E1 cells (day 7) (**Figure 4C**). The peak values of [Ca²^+^]_i_ elevation from basal levels were measured in undifferentiated and differentiating MC3T3-E1 cells. In differentiating cells, the BzATP-induced [Ca²^+^]_i_ rise was significantly attenuated by 5-BDBD treatment (**Figure 4D**).

BzATP-induced [Ca²^+^]_i_ increase was also significantly inhibited by P2X4 siRNA transfection in differentiating MC3T3-E1 cells (day 7). The peak [Ca²^+^]_i_ increases above basal levels were measured in differentiating MC3T3-E1 cells transfected with control or P2X4 siRNA (**Figures 4E, F**). The BzATP-induced [Ca²^+^]_i_ increase was significantly reduced in P2X4 siRNA-transfected cells compared with control siRNA-transfected cells (**Figure 4G**).

### 3.5 Enhancement of P2X4 currents in differentiating MC3T3-E1 cells

BzATP (100 μM)-induced P2X4 currents were measured as 5-BDBD (10 μM)-sensitive membrane current components in individual differentiating MC3T3-E1 cells using whole-cell patch-clamp recordings. Application of BzATP induced only slight changes in membrane currents in undifferentiated MC3T3-E1 cells (day 0) (**Figure 5A**), whereas differentiating MC3T3-E1 cells (day 7) exhibited a marked increase in current amplitude (**Figure 5B**). The BzATP-induced current response in differentiating MC3T3-E1 cells (day 7) was suppressed by co-application of 5-BDBD (**Figure 5B**). The 5-BDBD-sensitive current displayed weak inward rectification, consistent with previous reports describing similar current-voltage relationships for P2X4 channels (**Figure 5C**) (Ramirez and Kunze, 2002; Tang et al., 2008). The cell capacitance of MC3T3-E1 cells at day 0 and day 7 was 56.5 ± 5.8 pF and 43.0 ± 3.4 pF, respectively. The amplitude of the 5-BDBD-sensitive current density at –120 mV in differentiating MC3T3-E1 cells was significantly larger than that in undifferentiated cells (**Figure 5D**).

**Figure 5:**
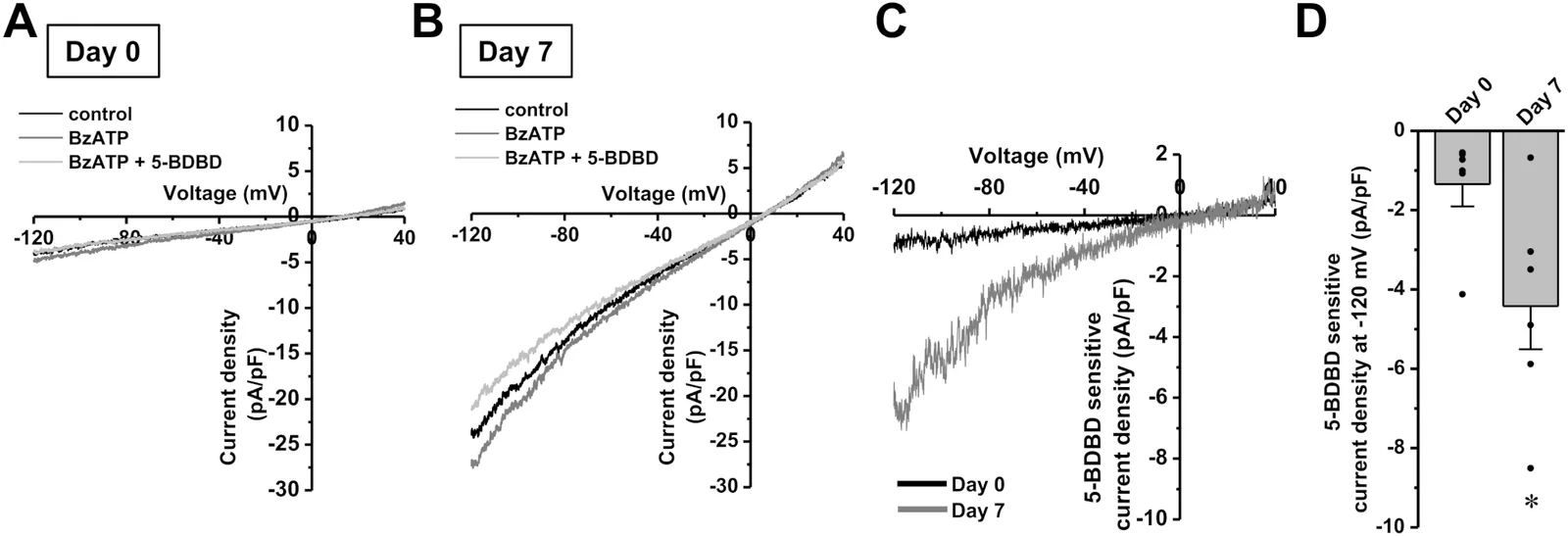
Upregulation of BzATP-induced P2X4 currents in differentiating MC3T3-E1 cells. **(A, B)** Membrane currents were recorded from differentiating MC3T3-E1 cells (day 0; **A**, day 7; **B**) using whole-cell patch-clamp recordings. I-V relationships were recorded in the absence and presence of 100 μM BzATP and 10 μM 5-BDBD. **(C)** Typical I–V relationships of the 5-BDBD–sensitive current component are shown as the difference between currents recorded in BzATP (gray line) and BzATP plus 5-BDBD (light gray line) in panels A, B. **(D)** The values of 5-BDBD-sensitive current density at −120 mV in differentiating MC3T3-E1 cells (day 0 and day 7) are summarized (n = 8 independent culture preparation per group, *P < 0.05 vs. vehicle).

### 3.6 Subcellular localization of P2X4 in differentiating MC3T3-E1 cells

To elucidate the mechanisms underlying P2X4 activity changes during osteoblast differentiation, we examined P2X4 protein expression in undifferentiated (day 0) and differentiating (day 7) MC3T3-E1 cells. Western blot analysis revealed no significant differences in total P2X4 protein expression between the two groups (**Figure 6A**). In contrast, analysis of the plasma membrane fraction revealed a significant increase in P2X4 protein expression in differentiating cells compared with undifferentiated cells (**Figure 6B**).

**Figure 6:**
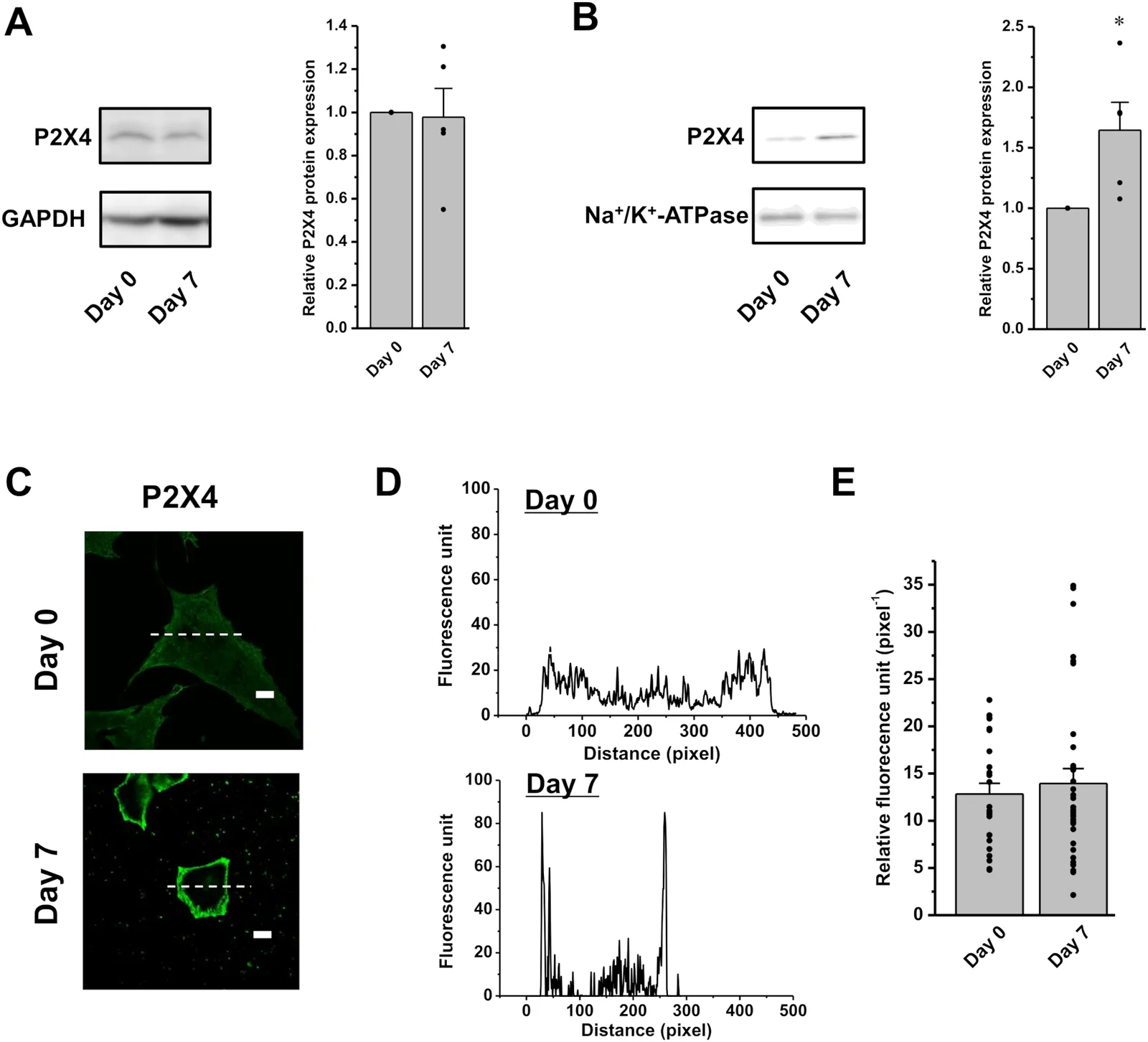
Protein expression and subcellular distribution of P2X4 in differentiating MC3T3-E1 cells. **(A)** Western blot analyses of P2X4 protein expression in MC3T3-E1 cells. Whole cell lysates from undifferentiated (day 0, lane 1) and differentiating (day 7, lane 2) MC3T3-E1 cells were immunoblotted with anti-P2X4 (top) and anti-GAPDH (bottom) on the same membrane. Data are summarized as the optical density of P2X4 and GAPDH band signals. Protein expression of P2X4 in the day 0 control was expressed as 1.0 after optical density of the P2X4 signal was normalized to that of the GAPDH signal (n = 5 independent culture preparation per group, p > 0.05 vs. day 0). **(B)** Western blot analyses of P2X4 protein expression in MC3T3-E1 cells. Plasma membrane fractions from undifferentiated (day 0, lane 1) and differentiating (day 7, lane 2) MC3T3-E1 cells were immunoblotted with anti-P2X4 (top) and anti-Na^+^/K^+^-ATPase (bottom) on the same membrane. Data are summarized as the optical density of P2X4 and Na^+^/K^+^-ATPase band signals. Protein expression of P2X4 in the day 0 control was expressed as 1.0 after optical density of the P2X4 signal was normalized to that of the Na^+^/K^+^-ATPase signal (n = 5 independent culture preparation per group, p < 0.05 vs. day 0). **(C)** Representative images of immunofluorescence detection of P2X4 in single undifferentiated (day 0, upper) and differentiating (day 7, lower) MC3T3-E1 cells. The scale bar is 10 μm. **(D)** The fluorescence profiles on the dotted line in panel **(C)** are shown in undifferentiated (day 0) and differentiating (day 7) MC3T3-E1 cells. **(E)** Fluorescence intensity normalized to cell area (intensity per pixel) in undifferentiated (day 0) and differentiating (day 7) MC3T3-E1 cells was quantified. Five independent experimental preparations were analyzed, with 4–7 cells measured per preparation. A total of 25 cells at day 0 and 33 cells at day 7 were analyzed. No significant difference was observed between the two groups (p > 0.05).

Subcellular localization of P2X4 was visualized using laser scanning confocal fluorescence microscopy after dissociation of the cells into single cells. In differentiating cells, P2X4 signals were localized along the plasma membrane, whereas they were distributed intracellularly in undifferentiated cells (**Figures 6C, D**). It has been reported that osteoblast differentiation induces morphological changes. Consistent with a previous report, the average cell area in differentiating MC3T3-E1 cells was 48.3 ± 3.8% of that in undifferentiated cells (p < 0.01; **Figures 6D, E**) (Itoh et al., 2021). Quantification of P2X4 signal intensity per cell in confocal images revealed no significant difference between undifferentiated and differentiating cells (**Figure 6E**). These findings indicate that the observed changes in P2X4 activity are likely attributable to subcellular localization rather than altered total protein expression.

### 3.7 Functional coupling between Panx3 and P2X4 in differentiating MC3T3-E1 cells

To elucidate whether P2X4 activation via Panx3-mediated ATP signaling plays a critical role in osteoblast differentiation, we examined the combined effects of a Panx3 inhibitor, a P2X agonist, and a P2X4 inhibitor on Alpl mRNA expression in differentiating MC3T3-E1 cells (day 7). As shown in **Figure 2A**, CBX (100 μM) markedly suppressed Alpl mRNA expression. This reduction was restored by co-treatment with BzATP (100 μM). Furthermore, additional treatment with P2X4 inhibitors (5 μM PSB and 10 μM 5-BDBD) significantly decreased Alpl mRNA expression (**Figure 7A**).

**Figure 7:**
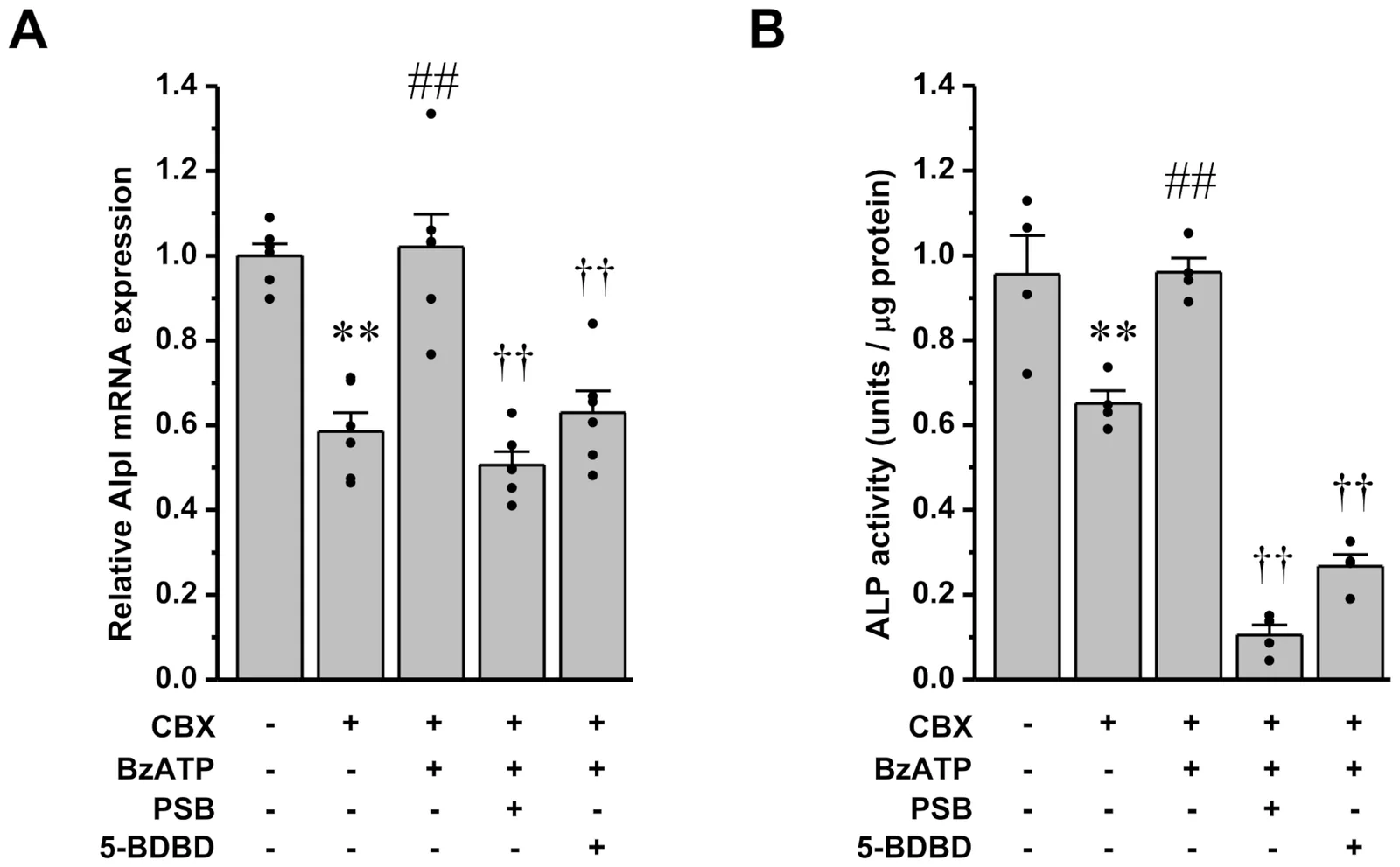
Functional coupling between Panx3 and P2X4 in differentiating MC3T3-E1 cells. **(A)** Real-time PCR for Alpl in differentiating MC3T3-E1 (day 7) co-treated with combinations of CBX (100 μM), BzATP (100 μM), PSB (5 μM) and/or 5-BDBD (10 μM) for 7 days. The expression level of Alpl mRNA was normalized to that in the vehicle control group (n = 6 for each group, ** p < 0.01 vs. CBX^−^BzATP^−^PSB^-^5-BDBD^−^, ## p < 0.01 vs. CBX^+^BzATP^−^PSB^−^5-BDBD^−^, †† p < 0.01 vs. CBX^+^BzATP^+^PSB^−^5-BDBD^−^). **(B)** ALP activity of differentiating MC3T3-E1 cells (day 7) was measured in the absence or presence of CBX (100 μM), BzATP (100 μM), PSB (5 μM) and/or 5-BDBD (10 μM) for 7 days (n = 6 for each group, ** p < 0.01 vs. CBX^−^BzATP^−^PSB^−^5-BDBD^−^, ## p < 0.01 vs. CBX^+^BzATP^−^PSB^−^5-BDBD^−^, †† p < 0.01 vs. CBX^+^BzATP^+^PSB^−^5-BDBD^−^).

To assess whether changes in Alpl mRNA expression corresponded to ALP enzyme activity, ALP activity was measured in differentiating cells (day 7). As shown in **Figure 2B**, CBX (100 μM) markedly suppressed ALP activity. This reduction was restored by co-treatment with BzATP (100 μM). Furthermore, additional treatment with P2X4 inhibitors (5 μM PSB and 10 μM 5-BDBD) significantly decreased ALP activity (**Figure 7B**).

### 3.8 Contribution of Panx3 and P2X4 to osteoblast differentiation and calcification in mouse bone marrow stromal cells

To investigate the role of Panx3 and P2X4 in the calcification of mouse bone marrow-derived osteoblasts, bone marrow stromal cells (BMSCs) were cultured in differentiation medium for 7 days with Panx3 or P2X4 inhibitors and then stained with Alizarin Red S to visualize calcium deposition (**Figure 8A**). Quantitative analysis revealed a significant increase in the amount of calcium deposition in differentiating BMSCs (day 7) (vehicle) compared with normal BMSCs (day 7) (vehicle). Treatment with CBX (100 μM) or 5-BDBD (5 and 10 μM) significantly attenuated this increase (**Figure 8B**). ALP activity was significantly higher in differentiating cells than in normal cells, and this increase was suppressed by 100 μM CBX and 5 μM 5-BDBD (**Figure 8C**). To further examine the effects of Panx3 and P2X4 inhibition on osteoblast differentiation, we examined Runx2 mRNA expression under the same experimental conditions. Runx2 expression was significantly increased in differentiating cells compared with in normal cells, whereas this increase was significantly attenuated by treatment with CBX (100 μM) or 5-BDBD (5 μM) (**Figure 8D**). To evaluate the contribution of Panx3 and P2X4 to calcification, we examined the effects of siRNA-mediated knockdown of these genes. In differentiating cells, both calcification and ALP activity were markedly increased in differentiating cells transfected with control siRNA compared with normal cells transfected with control siRNA, and these increases were significantly suppressed by knockdown of Panx3 or P2X4 (**Figures 8E–G**). Similarly, Runx2 mRNA expression was significantly increased following osteogenic differentiation, and this increase was significantly suppressed by knockdown of Panx3 or P2X4 (**Figure 8H**).

**Figure 8:**
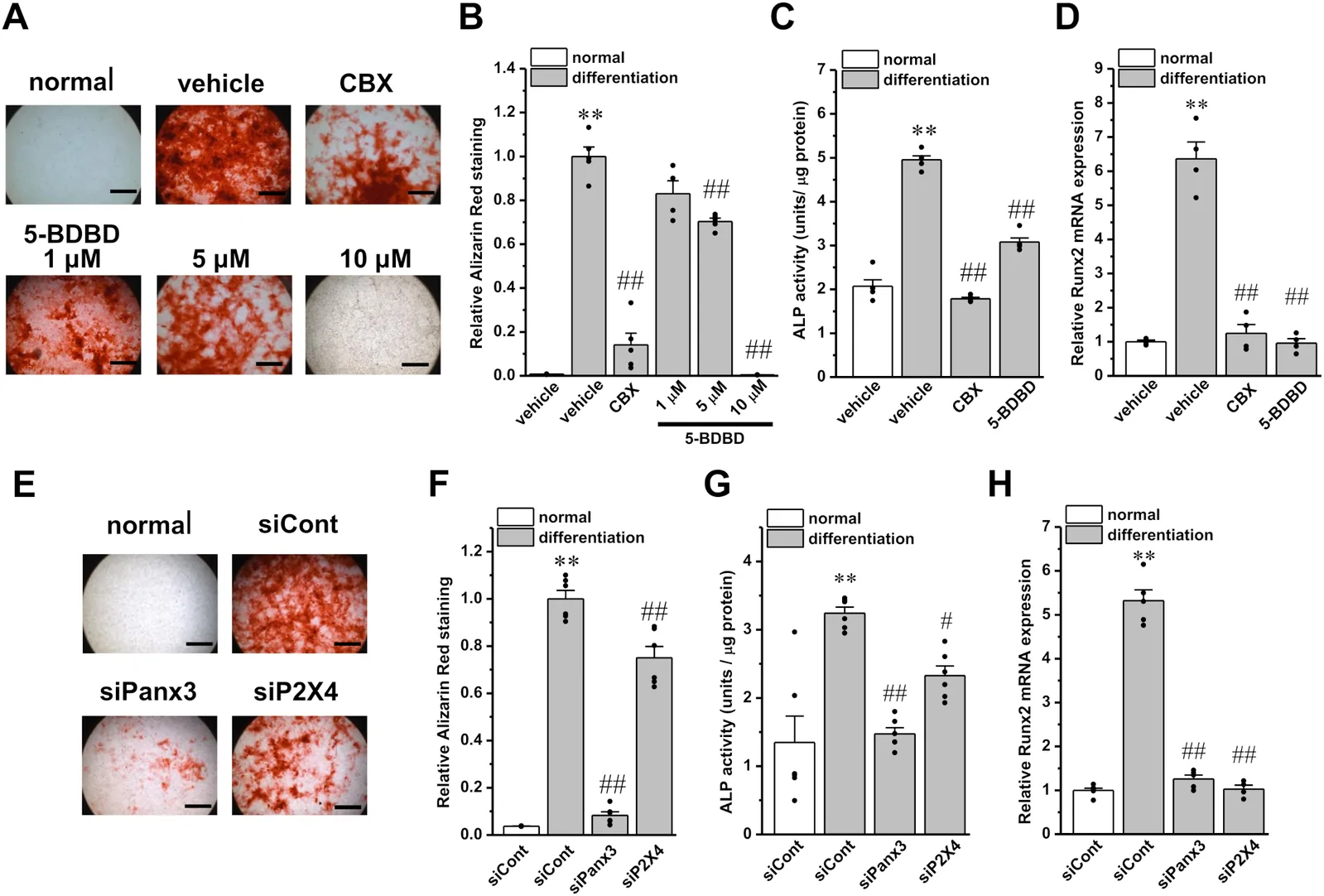
Contribution of Panx3 and P2X4 to osteoblast differentiation of mouse bone marrow stromal cells (BMSCs). **(A)** Representative images of Alizarin Red S-stained BMSCs in normal (day 7) or differentiation medium (day 7) with CBX (100 μM) and 5-BDBD (1, 5, and 10 μM). The scale bar is 500 μm. **(B)** Summarized data from experiments shown in panel **(A)** (n = 4–5 for each group, ** p < 0.01 vs. normal-vehicle, ## p < 0.01 vs. differentiation-vehicle). **(C)** ALP activity of BMSCs in normal (day 7) or differentiation medium (day 7) was measured in the absence or presence of CBX (100 μM) and 5-BDBD (5 μM) (n = 5 for each group, ** p < 0.01 vs. normal-vehicle, ## p < 0.01 vs. differentiation-vehicle). **(D)** Real-time PCR analysis of Runx2 mRNA expression in BMSCs cultured in normal medium or differentiation medium for 7 days in the absence or presence of CBX (100 μM) or 5-BDBD (5 μM) (n = 4 for each group). Runx2 mRNA expression was normalized to that in the normal-vehicle group. ** p < 0.01 vs. normal-vehicle, ## p < 0.01 vs. differentiation-vehicle. **(E)** Representative images of Alizarin Red S-stained BMSCs transfected with siCont, siPanx3, and siP2X4 in normal (day 7) or differentiation medium (day 7). The scale bar is 500 μm. **(F)** Summarized data from experiments shown in panel **(E)** (n =6 for each group, ** p < 0.01 vs. normal-siCont, ## p < 0.01 vs. differentiation-siCont). **(G)** ALP activity of BMSCs transfected with siCont, siPanx3, and siP2X4 was measured in normal (day 7) or differentiation medium (day 7) (n = 6 for each group, ** p < 0.01 vs. normal-siCont, #; ## p < 0.05; 0.01 vs. differentiation-siCont). **(H)** Real-time PCR analysis of Runx2 mRNA expression in BMSCs transfected with siCont, siPanx3, and siP2X4 in normal (day 7) or differentiation medium (day 7) (n = 5 for each group). Runx2 mRNA expression was normalized to that in the normal-siCont group. ** p < 0.01 vs. normal-siCont, ## p < 0.01 vs. differentiation-siCont.

### 3.9 Effects of Panx3 and P2X4 inhibition on endochondral ossification of mouse metatarsals

To further explore the effect of Panx3 and P2X4 on endochondral bone formation, murine metatarsals were isolated and cultured for 7 days in the absence or presence of CBX (100 μM) or PSB (3 μM). Metatarsals were imaged daily from day 1 to day 7, and the areas of the entire metatarsal as well as the mineralized regions were quantified (**Figures 9A, D**).

**Figure 9:**
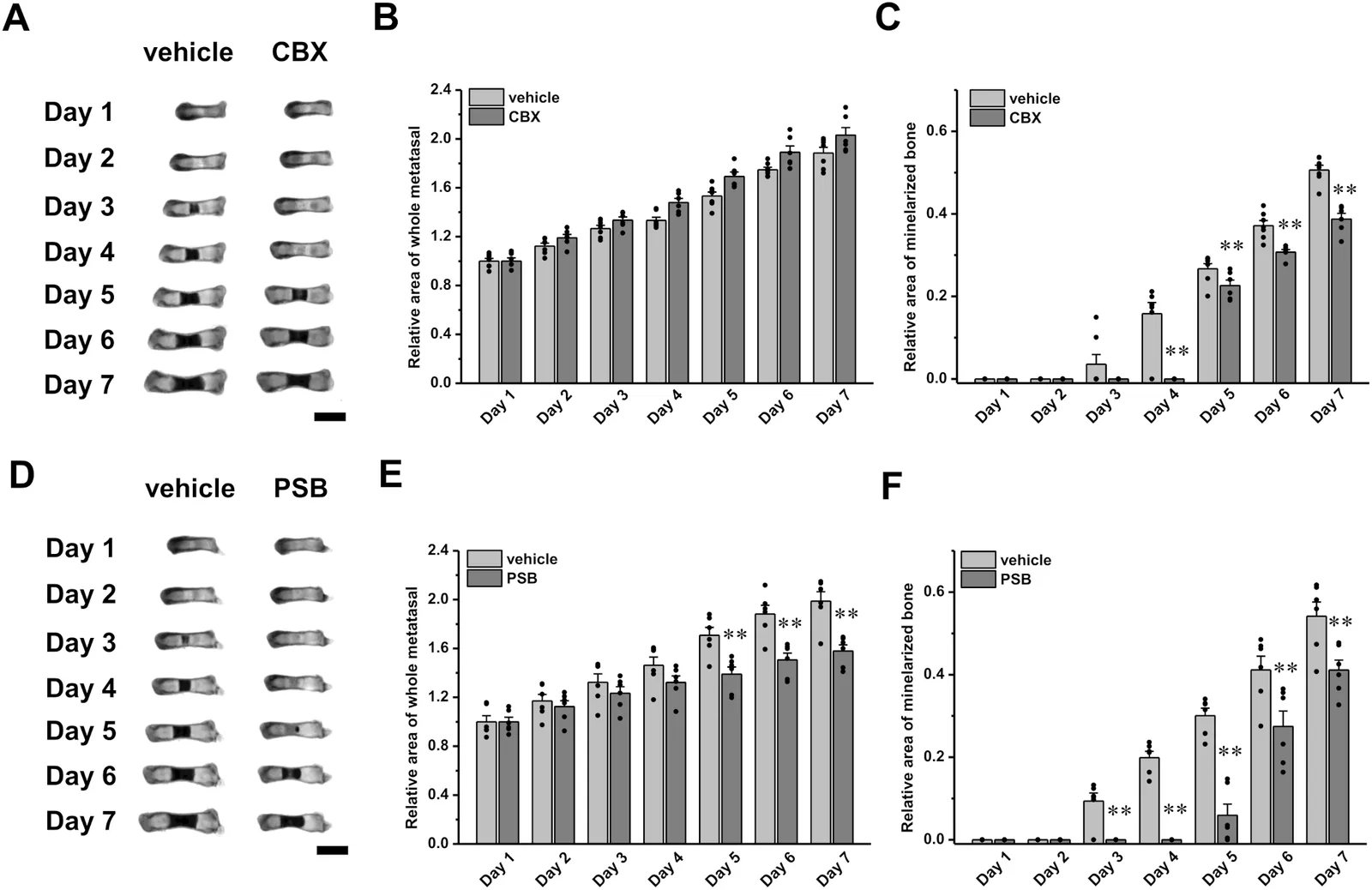
Effects of Panx3 and P2X4 inhibition on growth and mineralization of E15.5 mouse metatarsals. **(A, D)** Serial images of the third metatarsals with or without CBX (100 μM) **(A)** and PSB (3 μM) **(D)** at day 1 to day 7 of culture. Total bone area and central mineralization zone area were measured. The scale bar is 500 μm. **(B, E)** Each total bone area was normalized to that at day 1 in the absence or presence of CBX **(B)** and PSB **(E)** (CBX: n = 7, PSB: n = 6, ** p < 0.01 vs. vehicle). **(C, F)** The relative area of the central mineralized bone to total bone area with or without CBX **(C)** and PSB **(F)** (CBX: n = 7, PSB: n = 6, ** p < 0.01 vs. vehicle).

Throughout the 7-day culture period, no differences in the total metatarsal area were observed between the vehicle-treated group and the CBX-treated groups (**Figure 9B**). In contrast, the total metatarsal area was decreased in the PSB-treated group compared with the vehicle-treated group (**Figure 9E**). After 3 days of culture, endochondral ossification was observed in metatarsals treated with vehicle; however, no apparent calcification was detected in metatarsals treated with either CBX or PSB at this time point. Metatarsals treated with CBX or PSB showed the onset of endochondral ossification from day 5 of culture, but the mineralized area remained significantly smaller than that of the vehicle-treated group at day 7 (**Figures 9C, F**).

## 4 Discussion

The regulatory effect of ATP stimulation on osteoblast function has been shown to vary depending on the differentiation stage of the cells and the magnitude of the stimulus. Increasing evidence indicates that ATP signaling plays a pivotal role in modulating osteoblast activity, in part through the regulation of [Ca²^+^]_i_ (Grol et al., 2013; Orriss et al., 2013; Techatharatip et al., 2018; Gao et al., 2023). Our previous studies also demonstrated that the increase in [Ca²^+^]_i_ resulting from membrane hyperpolarization via Ca^2+^-activated K^+^ channel (K_Ca_3.1) activation plays a critical role in regulating osteoblast proliferation and differentiation (Kito et al., 2020; Kito et al., 2023). The [Ca²^+^]_i_ signaling in osteoblasts is known to facilitate osteoblast differentiation by modulating gene expression and enzymatic activity (Kito and Ohya, 2021). The present study suggests that functional coupling between Panx3 and P2X4 formed an ATP-dependent signaling mechanism that regulated osteoblast differentiation by modulating [Ca²^+^]_i_. Potential downstream targets of this P2X4-mediated Ca²^+^ influx may include the Calcineurin/NFATc1 pathway or CaMKII signaling, both of which are known to enhance the expression of osteogenic transcription factors such as Runx2 and Osterix (Koga et al., 2005; Choi et al., 2013).

When MC3T3-E1 cells were cultured in osteogenic differentiation medium, a time-dependent increase in the expression of osteoblastic markers, including Alpl and BSP, was observed (**Figures 1A, B**). In contrast, Runx2 expression did not differ significantly among days 0, 7, 14, and 21 (**Supplementary Figure S1**). Runx2 is primarily involved in the early stages of osteoblast differentiation, and is expressed in MC3T3-E1 pre-osteoblasts prior to their maturation (Luttrell et al., 2019). Previous studies using MC3T3-E1 cells have shown that Runx2 expression increases during the early stages of differentiation and subsequently reaches a plateau (Wu et al., 2014) or declines (Pregizer et al., 2008). Thus, the lack of a significant change in Runx2 expression in the present study may reflect its temporal expression pattern during osteogenic differentiation. To assess the potential role of ATP signaling in the early phase of osteoblast differentiation, the expression of pannexin and P2X receptor mRNAs was evaluated seven days after the induction of osteoblast differentiation. Notably, Panx3 and P2X4 mRNA levels were markedly high at this time point (**Figures 1E, F**). In contrast, the mRNA expression levels of P2Y receptor subtypes were relatively low compared with that of P2X4, suggesting a potentially limited contribution of P2Y-mediated signaling under our experimental conditions (**Figure 1G**). Seven days after the induction of osteogenic differentiation, quantitative analysis showed that the mRNA expression levels of Panx3 and P2X4 were elevated approximately sevenfold relative to undifferentiated controls (**Supplementary Figure S3**). Besides pannexins, vesicular nucleotide transporters (VNUT) and anion transport proteins such as progressive ankylosis protein (ANK) have also been reported to contribute, either directly or indirectly, to ATP release in osteoblasts (Minashima et al., 2017; Inoue et al., 2020). Although VNUT and ANK mRNA expression levels also increased following osteogenic differentiation, the extent of upregulation was relatively modest compared to that of Panx3 (**Supplementary Figure S4**). These findings suggest that further investigations are needed to clarify the regulatory roles of VNUT- and ANK-mediated ATP signaling in osteoblasts.

To elucidate the contribution of Panx3 to osteoblast differentiation of MC3T3-E1 cells, we investigated the effects of pannexin inhibition on ALP expression and activity. Both CBX treatment and Panx3 siRNA-mediated knockdown decreased Alpl mRNA expression and enzymatic activity in differentiating MC3T3-E1 cells (**Figures 2A–E**). Moreover, pharmacological and siRNA-mediated knockdown of Panx3 significantly suppressed the extracellular ATP levels (**Figures 2H, I**). Several studies have demonstrated that osteoblast differentiation promoted by mechanically induced ATP release is significantly attenuated by the administration of the ATP-hydrolyzing enzyme apyrase in human mandibular-derived osteoblast precursors (Sindhavajiva et al., 2018) and human bone marrow mesenchymal stem cells (Sun et al., 2013). Consistent with these findings, apyrase treatment significantly suppressed Alpl mRNA expression during the differentiation of MC3T3-E1 cells (**Figure 2J**). These data support the role of Panx3-mediated ATP signaling in facilitating osteoblast differentiation.

To clarify the involvement of P2X4 in osteoblast differentiation of MC3T3-E1 cells, we investigated the effects of P2X inhibition on ALP expression and activity. Both pharmacological blockade and siRNA-mediated knockdown of P2X4 decreased Alpl mRNA expression and enzymatic activity in differentiating MC3T3-E1 cells (**Figure 3**). This finding is consistent with a previous report indicating that pharmacological inhibition of P2X4 suppressed osteoblast differentiation in primary osteoblasts isolated from Sprague-Dawley rats (Orriss et al., 2023). Furthermore, functional analysis of P2X4 demonstrated that osteogenic differentiation enhanced the BzATP-induced Ca²^+^ influx and membrane currents mediated by P2X4, which were attenuated by treatment with the P2X4-specific inhibitor 5-BDBD (**Figures 4** and **5**). In addition, the BzATP-induced Ca²^+^ influx was significantly reduced in P2X4 knockdown MC3T3-E1 cells (**Figures 4E–G**). These results provide the first electrophysiological evidence demonstrating the functional expression of P2X4 in osteoblasts, suggesting that P2X4 activation plays a critical role in the regulation of osteoblast differentiation.

To explore the mechanism underlying enhanced P2X4 activity observed during osteoblast differentiation, we examined P2X4 protein expression and localization. Although total P2X4 protein levels remained largely unchanged throughout the differentiation process, immunocytochemical analysis revealed a marked translocation of P2X4 from the cytoplasm to the plasma membrane in differentiating MC3T3-E1 cells (**Figure 6**). P2X4 receptors have been shown to localize to lipid raft microdomains, defined in part by the presence of flotillin family proteins (Allsopp et al., 2010). To investigate whether P2X4 was localized to lipid rafts in differentiating MC3T3-E1 cells, fractionation analysis was performed; however, P2X4 was not detected in the raft fractions marked by Flotillin-2 (FLOT2) expression (**Supplementary Figure S5**). The observed reduction in cell area during differentiation (**Figure 6E**) effectively increases cell density, which likely narrows the interstitial space between neighboring osteoblasts. Although we did not directly measure the local ATP concentration in the intercellular spaces, this increased physical proximity suggests that higher cell density might facilitate more efficient Panx3-P2X4 signaling by creating a signaling microdomain.

To elucidate the roles of Panx3 and P2X4 under more physiologically relevant conditions, we examined the involvement of Panx3 and P2X4 in the osteogenic differentiation of BMSCs. Pharmacological inhibition of Panx3 and P2X4 led to a significant reduction in calcium deposition, ALP activity, and Runx2 mRNA expression during the osteogenic differentiation of BMSCs, suggesting that both molecules are critically involved in the maturation of bone marrow-derived osteoblasts (**Figure 8**). Furthermore, an ex vivo endochondral ossification model using isolated murine metatarsal bones demonstrated that the pharmacological inhibition of Panx3 and P2X4 significantly suppressed primary bone formation. These findings suggest that, beyond their roles in early osteogenic differentiation, Panx3 and P2X4 may also be critically involved in the maturation and mineralization of bone tissue during endochondral ossification (**Figure 9**). Interestingly, while Panx3 inhibition did not affect metatarsal bone elongation, P2X4 inhibition significantly suppressed longitudinal growth. These findings indicate that P2X4, but not Panx3, plays a prominent role in the regulation of endochondral bone elongation during skeletal development. Although previous studies have demonstrated that P2X4-mediated ATP signaling plays a role in prechondrogenic condensation, its specific function in differentiated chondrocytes during bone tissue formation remains poorly understood (Kwon, 2012). This highlights the need for further investigations to clarify the involvement of P2X4 in chondrocyte maturation and bone development.

Studies using Panx3 knockout mice have demonstrated that ubiquitous Panx3 deletion suppresses neonatal bone formation, whereas osteoblast-specific knockout does not significantly alter the bone phenotype (Caskenette et al., 2016; Yorgan et al., 2019). Additionally, P2X4 knockout mice have been reported to exhibit increased bone mass, contrary to initial expectations (Ellegaard et al., 2021). These discrepancies may be attributed to factors such as the influence of non-target tissues, the specific Cre recombinase employed, mouse strain, sex, and age. Furthermore, compensatory mechanisms involving related molecules such as Panx1 and P2X7 should also be considered. Therefore, careful interpretation is warranted when assessing the roles of Panx3 and P2X4 in bone homeostasis using mouse models. Taken together, our findings from osteoblasts provide valuable insights into the roles of Panx3 and P2X4 in the regulation of osteoblast function during bone formation *in vivo*.

In summary, our study demonstrates that Panx3 and P2X4 are functionally expressed in differentiating MC3T3-E1 cells. Notably, we provided the first electrophysiological characterization of P2X4-mediated membrane currents in osteoblast-lineage cells, revealing that their functional activity is significantly enhanced through the redistribution of the receptor from the cytoplasm to the plasma membrane during differentiation. Panx3-mediated ATP release activates these surface-expressed P2X4 receptors, inducing intracellular Ca²^+^ influx and subsequently promoting the progression of osteoblast differentiation. Furthermore, our results suggest that ATP signaling via the Panx3-P2X4 axis plays a critical regulatory role in the endochondral ossification of metatarsal bones. Although the upstream factors controlling Panx3 and P2X4 during osteogenesis remain to be identified, our findings from both cellular and ex vivo models highlight the essential role of the Panx3/P2X4 signaling pathway in osteoblast maturation and bone formation. These results provide novel insights into purinergic signaling mechanisms in bone biology and identify the Panx3/P2X4 axis as a potential molecular target for therapeutic strategies to promote osteogenesis.

## Data Availability

The raw data supporting the conclusions of this article will be made available by the authors, without undue reservation.
