# Supplementary material for: Pannexin 3-mediated adenosine triphosphate release from pre-osteoblasts promotes osteoblast differentiation through P2X4 receptor activation
Source: Front Physiol. 2026 Sep 21;17:1880201. doi: 10.3389/fphys.2026.1880201 (PMC13634857; doi:10.3389/fphys.2026.1880201)
Supplement: Supplementary file 1 [file DataSheet1.pdf]

Supplemental Figures

Supplemental Fig. S1

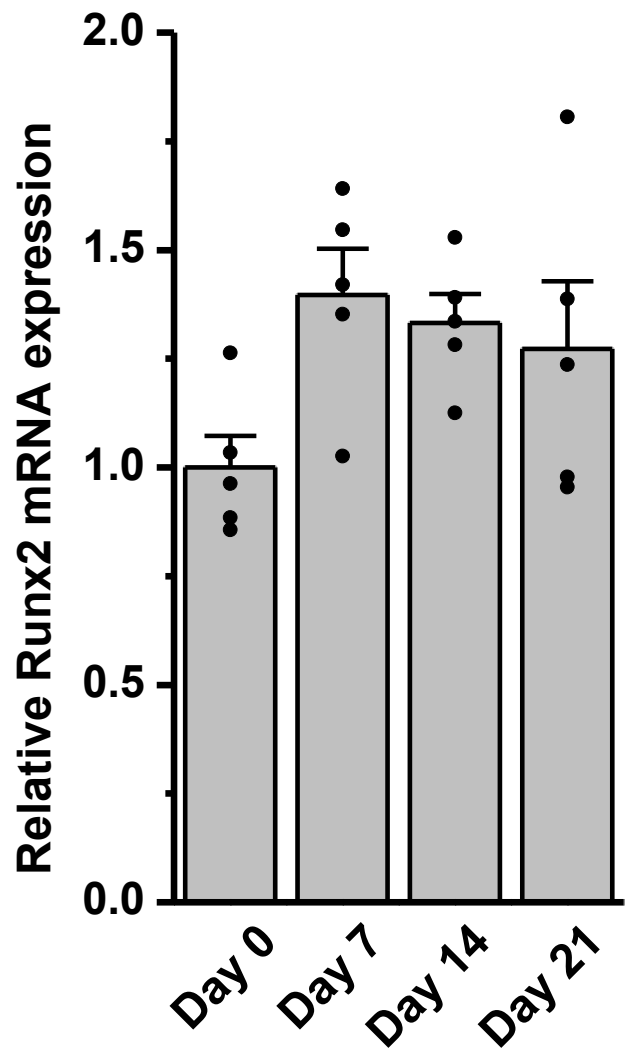

Figure S1 Time-dependent changes in Runx2 mRNA expression in differentiating MC3T3-E1 cells.

Real-time PCR for Runx2 in differentiating MC3T3-E1 (days 0, 7, 14, and 21) (n = 6 independent culture preparation per group). The expression levels of Runx2 mRNA were normalized to the expression levels at day 0.

Supplemental Fig. S2

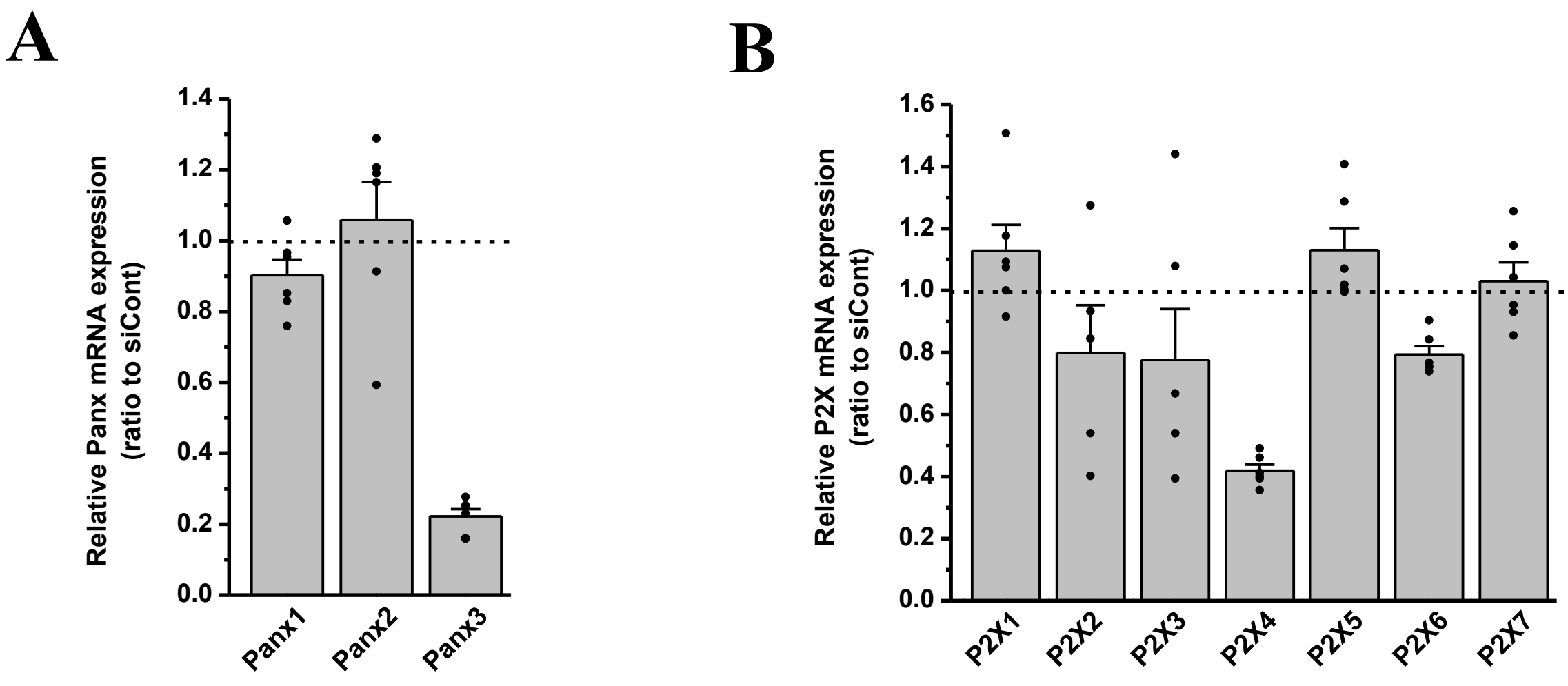

Figure S2 The effects of siRNA-mediated inhibition of Panx3 and P2X4 on the mRNA expression levels of other pannexin and P2X subtypes in MC3T3-E1 cells.

(A): Real-time PCR for pannexin subtypes (Panx1, Panx2, and Panx3) in differentiating MC3T3-E1 (day 7) transfected with Panx3 siRNA. The expression level of pannexin mRNA was normalized to that in the control siRNA (siCont) group (n = 6 for each). **(B):** Real-time PCR for P2X subtypes (P2X1-P2X7) in differentiating MC3T3-E1 (day 7) transfected with P2X4 siRNA. The expression level of P2X mRNA was normalized to that in the control siRNA (siCont) group (n = 6 for each).

Supplemental Fig. S3

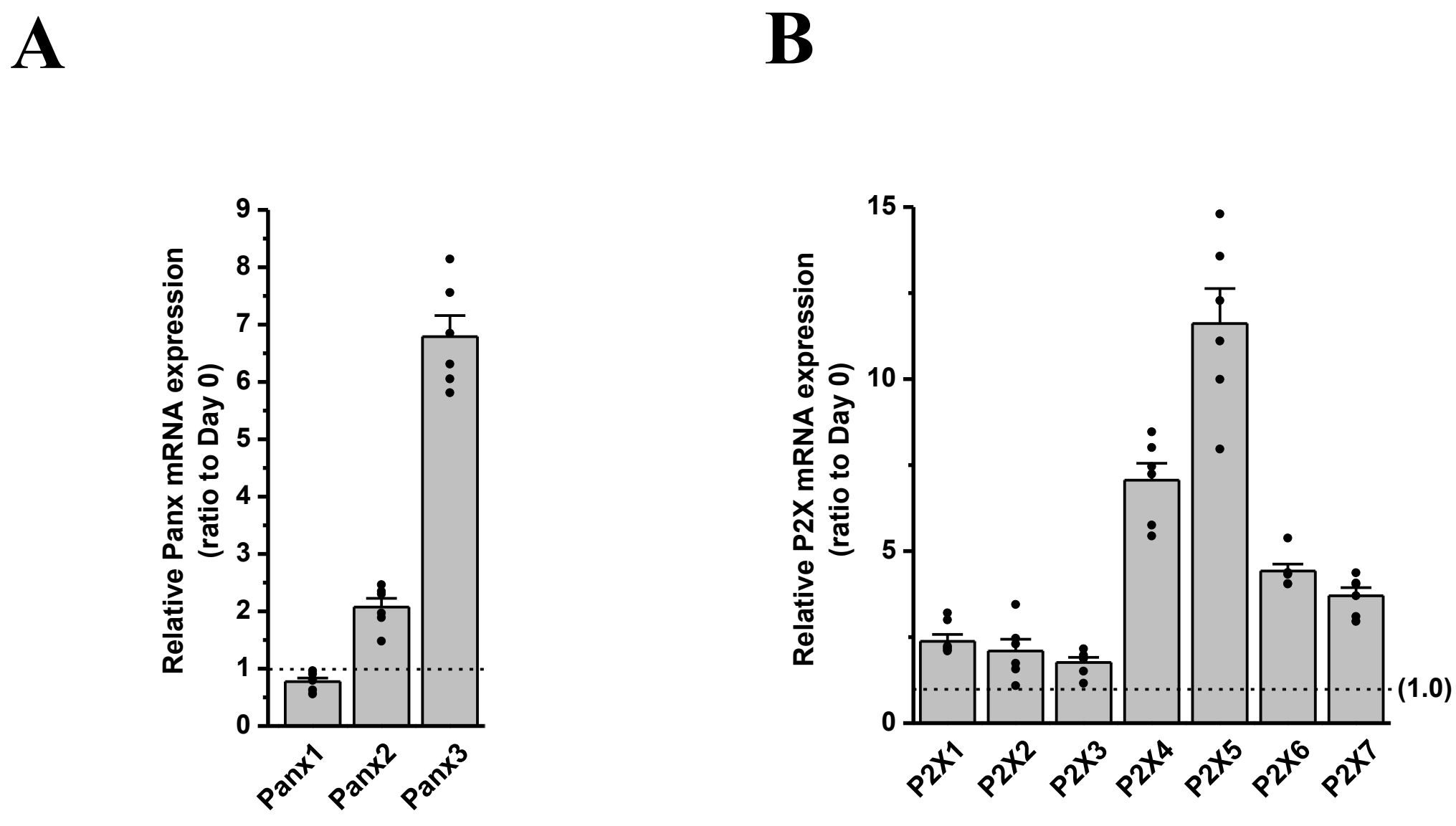

Figure S3 Upregulation of Pannexin 3 and P2X4 mRNA in differentiating MC3T3-E1 cells.

(A): Real-time PCR for pannexin subtypes in differentiating MC3T3-E1 (day 7). The mRNA expression levels of pannexin subtypes in MC3T3-E1 cells were normalized to endogenous GAPDH and expressed relative to the levels at day 0 (n = 6 for each). **(B):** Real-time PCR for P2X subtypes in differentiating MC3T3-E1 (day 7). The mRNA expression levels of P2X subtypes in MC3T3-E1 cells were normalized to endogenous GAPDH and expressed relative to the levels at day 0 (n = 6 for each).

Supplemental Fig. S4

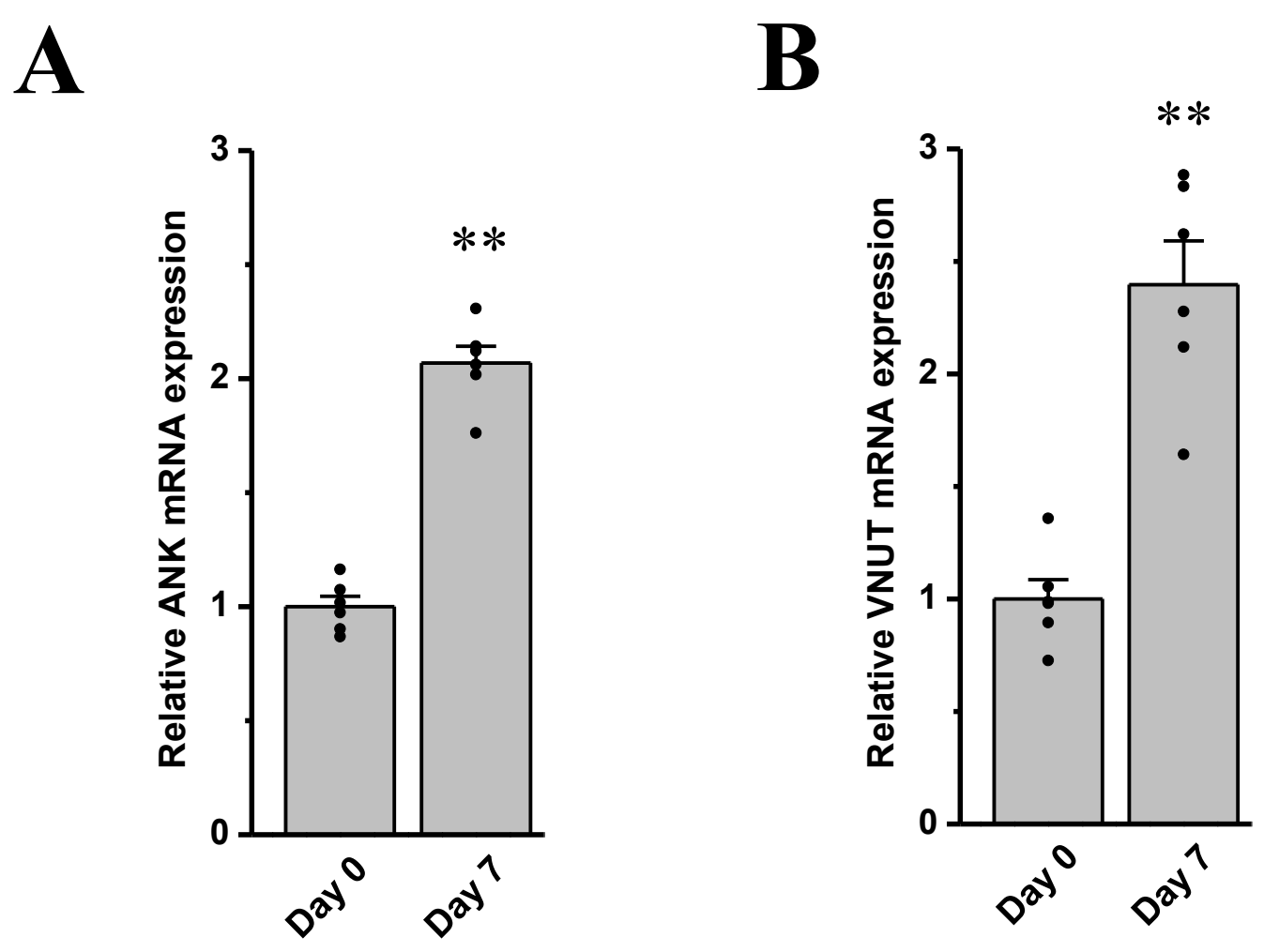

Figure S4 Changes in the expression levels of ANK and VNUT mRNA in differentiating MC3T3-E1 cells.

Real-time PCR for ANK **(A)** and VNUT **(B)** in differentiating MC3T3-E1 (day 7). The expression levels of ANK and VNUT mRNA were normalized to those at day 0 (n = 6 for each, ** $p < 0.01$ vs. Day 0).

Supplemental Fig. S5

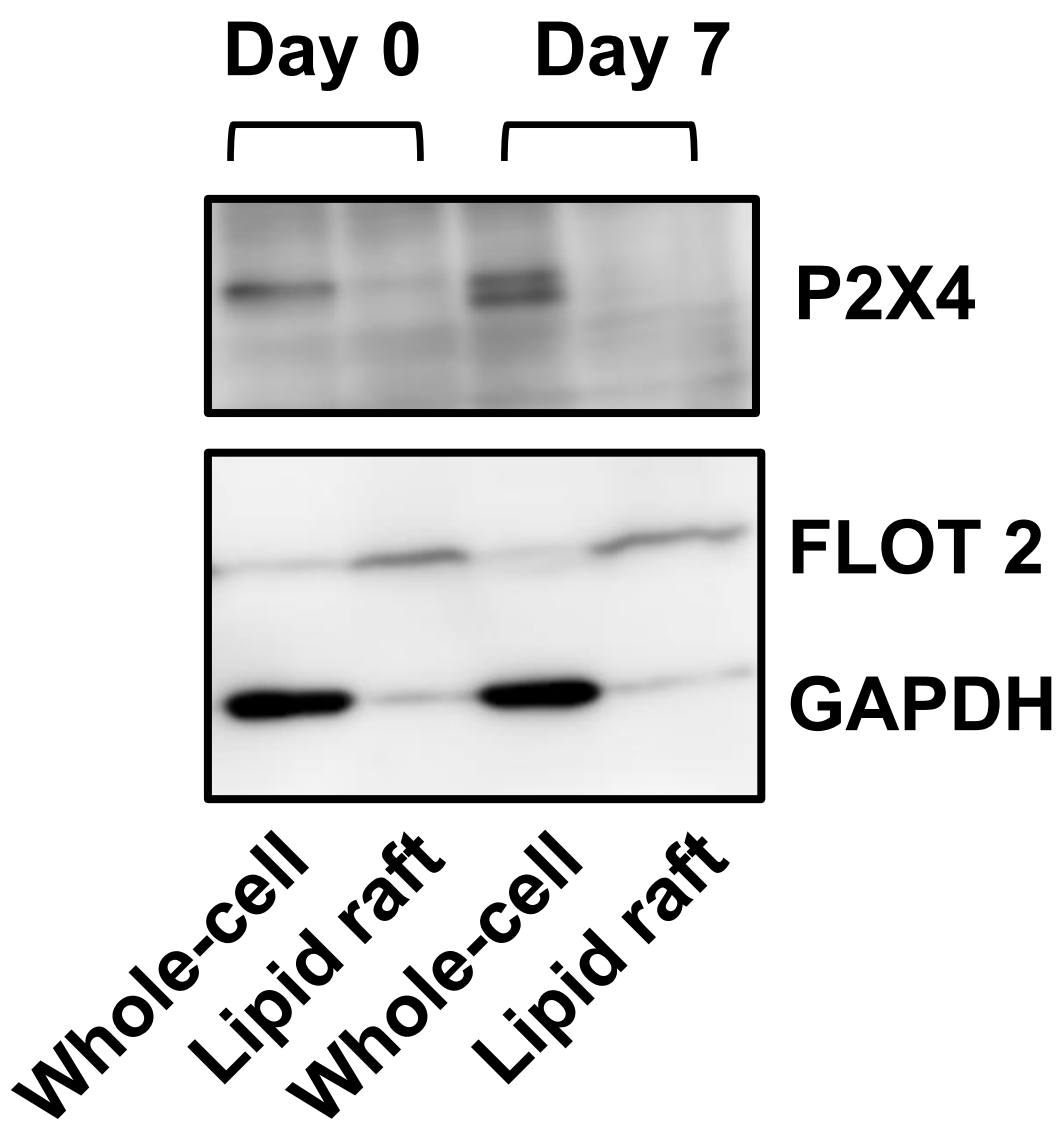

Figure S5 The protein expression of P2X4 in lipid raft domain of MC3T3-E1 cells

Whole-cell and lipid raft lysates were prepared from differentiating MC3T3-E1 cells (day 0 and day 7) using UltraRIPA kit for Lipid Raft. Protein lysates of MC3T3-E1 cells (10 μg/lane) were immunoblotted with anti-P2X4, anti-FLOT2, and anti-GAPDH antibodies on the same filter.
